# Saliva metabolomics: a non-invasive frontier for diagnosing and managing oral diseases

**DOI:** 10.1186/s12967-025-06587-z

**Published:** 2025-05-24

**Authors:** Xinyuan Zhao, Xu Chen, Ye Lu, Zihao Zhou, Pei Lin, Yunfan Lin, Shen Hu, Li Cui

**Affiliations:** 1 Stomatological Hospital, School of Stomatology, Southern Medical University, Guangzhou, 510280 Guangdong China; 2 School of Dentistry, Jonsson Comprehensive Cancer Center, California NanoSystems Institute, University of California, Los Angeles, Los Angeles, CA 90095 USA

**Keywords:** Oral diseases, Saliva metabolites, Metabolomics, Early diagnosis, Disease monitoring

## Abstract

Salivary metabolomics represents a powerful noninvasive approach for diagnosing, monitoring, and managing oral diseases, providing valuable insights into the metabolic alterations associated with conditions such as oral cancer, oral precancerous lesions, periodontal diseases, and dental caries. Through the comprehensive analysis of salivary metabolites, this methodology facilitates the identification of disease-specific biomarkers reflective of underlying pathophysiological processes, including inflammation, microbial dysbiosis, and metabolic reprogramming. Despite its promising clinical potential, several significant challenges remain, notably the difficulty in establishing direct associations between specific salivary metabolites and distinct disease mechanisms, considerable inter-individual variability, and the inherent complexity of the oral microenvironment. Furthermore, issues related to data interpretation complexity, technological constraints, and the necessity for rigorous clinical validation continue to impede its broader clinical adoption. Nevertheless, ongoing advancements in analytical technologies and bioinformatics approaches hold considerable promise for addressing these limitations, positioning salivary metabolomics as a transformative tool for precision diagnosis and personalized treatment in oral health care.

## Introduction

Early and accurate diagnosis of oral diseases—particularly oral cancer, periodontal disease, and dental caries—is critical for improving patient prognosis and guiding timely therapeutic interventions. However, many oral diseases progress insidiously and often present with non-specific or subclinical symptoms in the early stages, making timely detection and monitoring particularly challenging in routine clinical practice [1, 2]. Conventional diagnostic approaches, such as tissue biopsy and radiographic imaging, are often invasive and may fail to capture dynamic pathological changes, limiting their utility for early detection and longitudinal assessment. Consequently, there is an urgent need for reliable, non-invasive biomarkers that can detect disease onset, monitor progression, and evaluate treatment responses with high sensitivity and specificity. Such biomarkers could not only facilitate earlier intervention but also improve therapeutic decision-making, enhance patient outcomes, and reduce healthcare costs through more efficient disease management.

Saliva metabolomics offers a compelling alternative to traditional diagnostic techniques, particularly in its ability to capture the metabolic alterations associated with oral diseases. As a noninvasive and readily accessible biofluid, saliva reflects both local oral pathology and systemic physiological changes [3–6]. Unlike blood or tissue samples, which may not fully represent oral-specific processes, saliva metabolomics offers a comprehensive view of the biochemical activities within the oral cavity, including interactions between the oral microbiome, immune system, and host metabolism. The analysis of salivary metabolites can uncover critical insights into disease pathways, such as the onset of inflammation, microbial dysbiosis, and shifts in metabolic networks—all of which are essential for understanding the progression of oral pathologies. Additionally, saliva collection is painless and suitable for repeated, longitudinal sampling, making it ideal for tracking disease dynamics and treatment responses without the need for invasive procedures [3, 7, 8].

In this review, we explore the potential of saliva metabolomics for diagnosing and monitoring oral diseases, focusing on its capacity to identify biomarkers for early detection, disease progression, and therapeutic assessment. The advantages of using saliva as a diagnostic medium are examined, along with key methodologies in metabolomic profiling and emerging metabolite-based biomarkers associated with oral pathological conditions such as oral cancer, periodontal diseases, and caries. Additionally, challenges hindering the clinical application of saliva metabolomics are discussed, including the complex composition of saliva, individual variability, and the need for comprehensive clinical validation.

## The multifaceted advantages of saliva as a diagnostic tool for oral diseases

**Fig. 1:**
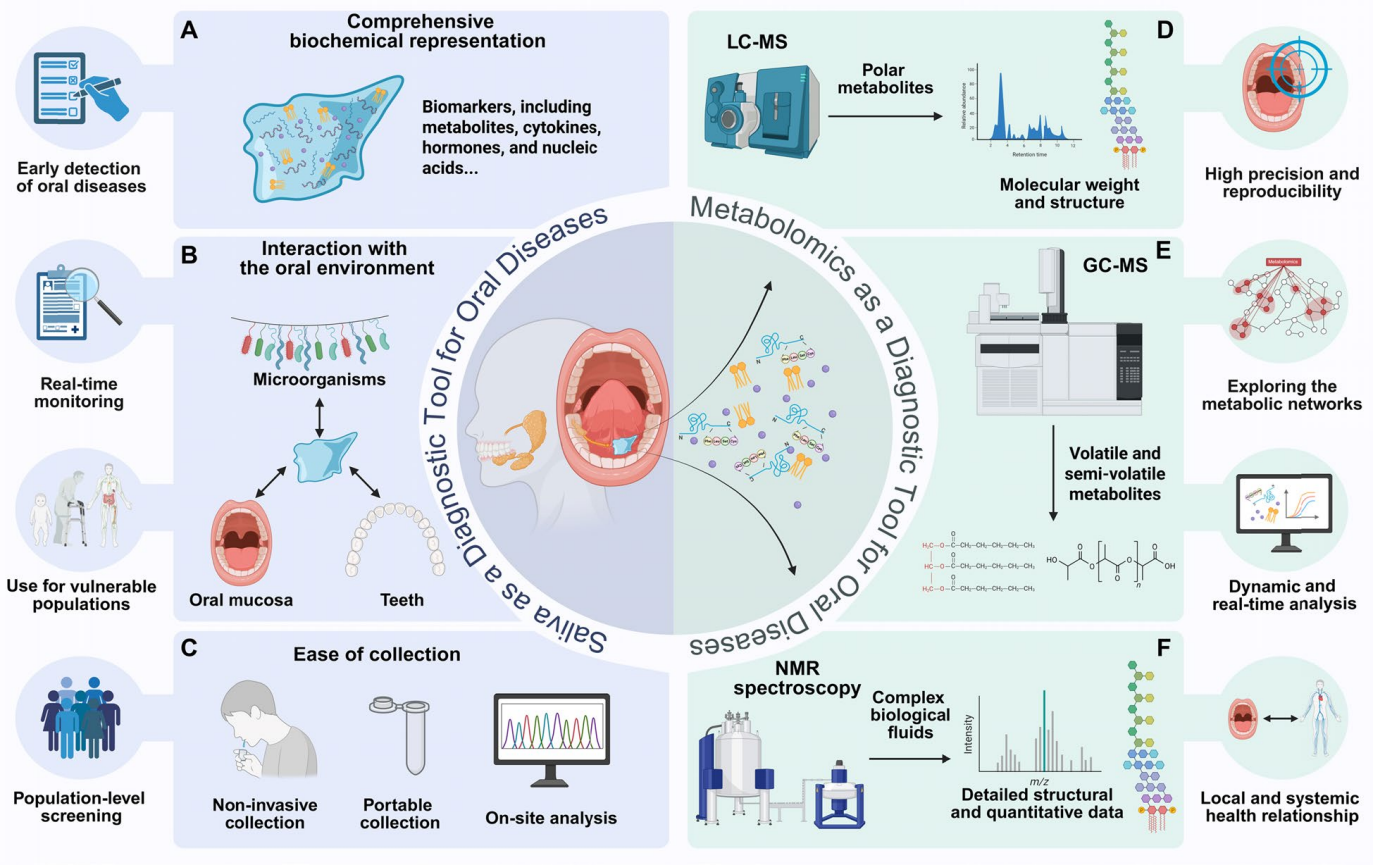
The multifaceted advantages of saliva and metabolomics as a diagnostic tool for oral diseases. **A–C** Saliva contains a wide range of biomarkers and interacts continuously with the oral microenvironment, providing a readily accessible, non-invasive medium for early disease detection, real-time monitoring, and large-scale screening. **D–F** Metabolomics, as a diagnostic approach, employs advanced techniques such as LC–MS, GC–MS, and NMR spectroscopy. Each method targets specific categories of metabolites, delivering high precision and reproducibility. These approaches enable the visualization of complex metabolic networks and facilitate dynamic monitoring of disease progression. Notably, it not only captures localized oral changes but also reflects systemic health

Saliva possesses distinct advantages as a diagnostic medium for oral diseases, surpassing traditional biofluids such as blood, tissue, and other body fluids in several key aspects [9]. Its unique interaction with the oral environment, coupled with its ease of collection and comprehensive biochemical representation, renders saliva a versatile and effective tool for understanding, diagnosing, and monitoring oral diseases [10]. Unlike blood, which reflects systemic changes diluted by various physiological processes, saliva directly interacts with the oral mucosa, teeth, and microorganisms, capturing localized metabolic and microbial changes with high specificity. This specificity facilitates the early detection of oral diseases at their point of origin, which may be missed by tissue biopsies limited to localized sampling sites [11]. Moreover, saliva’s capacity to capture the continuous interaction between host and microbial activity offers a holistic view of oral disease progression [12]. Additionally, saliva collection is painless, simple, and non-invasive, avoiding the discomfort and risks associated with blood draws or tissue biopsies. This makes it particularly suitable for vulnerable populations such as children, elderly patients, or individuals with compromised immune systems [13]. The non-invasive nature also enables frequent sampling for longitudinal studies or monitoring the efficacy of interventions, which is challenging with tissue biopsies or other invasive methods [14]. Moreover, saliva not only reflects local oral changes but also serves as a window into systemic health. It contains a rich array of biomarkers, including metabolites, cytokines, hormones, and nucleic acids, that link oral diseases with systemic conditions such as diabetes, cardiovascular disease, and autoimmune disorders [15–17] (Fig. 1).

Notably, saliva is a reservoir of diverse biomolecules, from small metabolites to DNA and RNA, offering a multi-dimensional diagnostic platform [18]. Unlike blood, which may require specialized anticoagulants or stabilization protocols, saliva is easier to process and store. Tissue samples, while rich in site-specific information, are invasive to obtain and often lack the dynamic metabolic data present in saliva. Saliva’s molecular diversity enables its application across a wide spectrum of oral diseases, from identifying early metabolic shifts in precancerous lesions to monitoring microbial dysbiosis in periodontitis [19]. Moreover, saliva’s composition dynamically reflects real-time physiological and pathological changes, making it an ideal medium for monitoring disease progression or treatment response [20]. For example, in patients undergoing non-surgical periodontal therapy, salivary metabolomics can track residual inflammation and metabolic restoration, offering insights into therapy success and areas requiring further intervention [21]. The simplicity and cost-effectiveness of saliva collection make it highly scalable for population-level screening and research. Portable collection devices and advances in on-site analysis tools, such as point-of-care diagnostics, enhance its accessibility in low-resource settings [22]. Furthermore, advancements in technologies such as high-throughput metabolomics, proteomics, and next-generation sequencing have significantly enhanced the diagnostic potential of saliva. Sophisticated analytical platforms like nuclear magnetic resonance (NMR) spectroscopy and liquid chromatography-mass spectrometry (LC–MS) enable the detection of subtle metabolic changes with high sensitivity. This precision is particularly valuable for early-stage disease detection, providing insights into conditions such as oral cancer and precancerous lesions [23].

To facilitate the clinical implementation of salivary metabolomics, standardized protocols for sample collection, processing, and analysis have been established to ensure reproducibility and minimize technical variability [24, 25]. Typically, unstimulated saliva is collected in the morning following a fasting period using low-binding tubes, immediately cooled, centrifuged to remove cellular debris, and stored at − 80 °C. Stable-isotope internal standards and pooled quality control samples are incorporated to monitor instrument performance and correct for batch effects, with data normalization strategies such as probabilistic quotient normalization or total ion current normalization applied in accordance with the Metabolomics Standards Initiative (MSI). Salivary metabolite profiling can be performed using both untargeted and targeted approaches. Untargeted platforms, such as hydrophilic interaction liquid chromatography coupled with high-resolution mass spectrometry and gas chromatography–mass spectrometry (GC–MS), enable broad-spectrum detection of metabolic features without prior selection [26]. Targeted methods, including liquid chromatography–tandem mass spectrometry in multiple reaction monitoring mode and proton NMR spectroscopy, offer high precision and quantification of predefined metabolites, making them well suited for clinical validation and multicenter studies [27]. These standardized methodologies provide a robust analytical foundation for the reliable identification of salivary biomarkers across diverse oral disease contexts.

## Overview of metabolomics

Metabolomics is the systematic study of all metabolites present in a biological sample, such as amino acids, lipids, sugars, and organic acids. It aims to provide a comprehensive analysis of the dynamic changes in metabolites, offering insights into biological processes, disease mechanisms, and drug responses. By capturing the full spectrum of metabolites within a biological system, metabolomics provides critical insights into the biochemical pathways that underpin health and disease, thereby supporting early disease detection, guiding therapeutic interventions, and advancing personalized medicine [28–30].

The core principle of metabolomics lies in the qualitative and quantitative analysis of metabolites using advanced analytical techniques. These techniques, such as LC–MS, GC–MS, and NMR spectroscopy, are employed to measure changes in metabolite levels across various biological conditions [31, 32]. LC–MS combines liquid chromatography, which separates metabolites based on their physical and chemical properties, with mass spectrometry, which provides information about the molecular weight and structure of the metabolites. This method is particularly effective for analyzing polar metabolites and those that are difficult to isolate [33–35]. GC–MS, on the other hand, is best suited for analyzing volatile and semi-volatile metabolites, such as fatty acids and organic acids, providing high resolution and sensitivity [36, 37]. NMR spectroscopy is a non-destructive method that can provide detailed structural and quantitative data about metabolites. Unlike mass spectrometry, NMR spectroscopy does not require prior sample separation, making it well-suited for the analysis of complex biological fluids. It is particularly advantageous for detecting high-abundance metabolites and for elucidating metabolic networks in living systems [38, 39].

Metabolomics can be categorized into targeted and untargeted approaches, each serving distinct purposes in metabolic analysis. Targeted metabolomics involves the analysis of a predefined set of metabolites, focusing on known biomarkers or specific metabolic pathways. This approach is characterized by its high precision and reproducibility, as it targets a limited number of metabolites, often using internal standards for accurate quantification. Targeted metabolomics is ideal for absolute quantification, providing precise concentration measurements of specific metabolites. It is particularly useful for hypothesis testing, disease monitoring, and tracking metabolic alterations associated with specific conditions or therapeutic interventions. Furthermore, this approach is well-suited for validating previously identified biomarkers and ensuring the consistency and reliability of findings across different samples and conditions [40–42]. In contrast, untargeted metabolomics entails the comprehensive and unbiased profiling of all detectable metabolites within a biological sample, without reliance on predefined selection criteria. This approach offers a holistic view of the metabolic landscape, making it particularly valuable for investigating the complexity of metabolic networks and uncovering previously unrecognized metabolic alterations associated with disease. Although it is less focused on absolute quantification, untargeted metabolomics provides exceptional discovery potential, yielding insights into novel disease mechanisms and facilitating the identification of new therapeutic targets [43–45]. One of the key characteristics of metabolomics is its high sensitivity and resolution. Techniques such as LC–MS and NMR can detect metabolites at very low concentrations, allowing for the identification of early-stage biochemical changes that may not be detectable by other methods [46]. Moreover, metabolomics provides a dynamic and real-time analysis of metabolic changes, making it ideal for monitoring disease progression, therapeutic responses, and environmental influences on metabolic processes. This dynamic nature distinguishes metabolomics from static analyses like genomics or proteomics, which provide valuable but more fixed snapshots of biological systems [47, 48]. Metabolomics also plays a critical role in understanding the relationship between local and systemic health. By analyzing metabolites in body fluids, it is possible to detect subtle shifts that reflect systemic changes or the impact of oral health on overall well-being. Importantly, high-throughput metabolomics enables large-scale studies that analyze multiple samples simultaneously, making it possible to discover biomarkers for early diagnosis, prognosis, and treatment monitoring. These studies can be used to explore disease mechanisms, identify therapeutic targets, and track changes in metabolic networks. Additionally, advances in bioinformatics and statistical modeling enable the extraction of meaningful insights from complex datasets, facilitating the identification of novel biomarkers and metabolic pathways that might otherwise remain undetected [49].

The reliability of salivary metabolomic biomarkers is influenced by both biological and technical variability. Biologically, individual factors such as age and sex affect baseline metabolic profiles due to differences in hormonal regulation, immune function, and salivary gland physiology. Circadian rhythms also contribute to diurnal variations in salivary composition, with measurable changes in amino acids, cortisol, and other metabolites observed throughout the day [50]. Dietary habits and recent food intake can result in short-term fluctuations in salivary metabolite levels, particularly affecting concentrations of glucose, lipids, and short-chain fatty acids [51]. In addition, systemic health conditions such as diabetes, cardiovascular diseases, and autoimmune disorders may alter salivary metabolite composition by modulating systemic inflammatory and metabolic responses [52]. Salivary flow rate, influenced by hydration status, medication use, and autonomic regulation, affects the dilution and concentration of metabolites. Furthermore, the oral microbiome can produce microbial-derived metabolites that vary between individuals and confound host-derived biomarker signals [53]. On the technical side, variability may arise from differences in sample collection timing, handling, storage conditions, and instrument performance. These biological and technical factors must be carefully controlled and accounted for to ensure the reproducibility and translational relevance of salivary metabolomic biomarkers [54]. Importantly, robust validation across large, well-characterized cohorts is essential to confirm the reproducibility, diagnostic accuracy, and clinical applicability of candidate salivary biomarkers.

## Salivary metabolomics for oral disease diagnosis and management

Oral diseases, ranging from precancerous lesions to chronic inflammation, often involve subtle metabolic and microbial shifts that are critical for early detection and intervention [55]. Salivary metabolomics, by analyzing the biochemical and microbial profiles in saliva, offers a non-invasive and highly sensitive approach to identifying biomarkers, monitoring disease progression, and assessing treatment efficacy [56, 57]. This method provides a dynamic tool for improving the understanding and management of oral health conditions, complementing traditional diagnostic techniques and offering new opportunities for clinical application (Table 1).

**Table 1:** Application of saliva metabolomics in the early detection and monitoring of oral diseases

Disease	Saliva Metabolites	Methods	Clinical application	Refs.
OLP	Acetate, methylamine, pyruvate, tyrosine	UPLC-HRMS	Early detectors of OLP	[60]
WSLs	Proline, glycine	16S rRNA-seq UPLC-MS/MS	Biomarkers of WSLs	[115]
OSCC	Kynurenic acid	UPLC-MS/MS	OSCC preventions and therapeutics	[69]
OSCC	Phosphatidic acid	LC–MS/MS	Early detection and cancer interception	[70]
OSCC	Choline, urea, 3-hydroxybutyric acid	CE-MS	Non-invasive diagnosis	[73]
TSCC	*N*-acetyl-d-glucosamine, l-pipecolic acid, l-carnitine	LC–MS	Disease monitoring and prognosis	[74]
OSCC	Malic acid, maltose, protocatechuic acid, catechol	GC–MS	Assistance in the identification of oral cancer salivary biomarkers	[75]
OSCC	Glycine, proline	NMR, LC–MS/MS, LC-Q-TOF	Early diagnosis	[76]
OSCC	1-Methylhistidinesphinganine-1-phosphate, ubiquinone	Q-TOF, LC–MS	Preventing malignant transformation of OLK	[78]
HNC	N1-acetylspermine	LC–MS/MS	Early diagnosis	[81]
HNSCC	Fucose, proline, 1,2-propanediol	NMR spectroscopy	Point-of-care platforms for HNSCC	[82]
OSCC	Indole-3-acetate, ethanolamine	Logistic regression	Non-invasive screening of OSCC and OLP	[84]
OSCC	Decanedioic acid, l-proline, pentanoic acid	GC–MS	Early diagnosis and prediction	[85]
Periodontitis	Tryptophan, phenylalanine	CPSI-MS	Preclinical screening of SP	[88]
Periodontitis	Ethanol, taurine, isovalerate, butyrate, glucose	1H-NMR spectroscopy	Periodontal screening, detection, and monitoring	[89]
AgP	Pyruvate, lactate, proline, phenylalanine, tyrosine	NMR spectroscopy	Distinguishing chronic and aggressive periodontitis	[90]
Periodontitis	Phenylacetate	UHPLC-MS/MS	Early intervention in the initial stage of periodontitis	[91]
Periodontitis	2-Pyrrolidineacetic acid, butyrylputrescine	UPLC-MS/MS	For self-screening of periodontitis	[92]
Generalized periodontitis	Lactate, pyruvate, tyrosine	NMR spectroscopy	Using to monitor treatment stages	[21]
Periodontitis	Tryptophan, glutathione	16s rRNA-seq	Potential tool in the diagnosis and prognosis evaluation of periodontitis	[94]
Active carious	Taurine, mannose	NMR spectroscopy	Advancing personalized approaches to dental caries prevention	[99]
Caries	Histamine, l-histidine, succinate	LC–MS/MS	Biomarkers for monitoring the treatment effects of dental caries	[100]
SECC	2-benzylmalate, epinephrine, 3-Indoleacrylic acid	16S rRNA seq	Future strategies for personalized caries	[101]
BMS	Paraxanthine, theophylline	UPLC-Q-TOF–MS	Potential therapeutic approaches for BMS	[104]
Primary BMS	L-dopa, l-tyrosine, tyramine	LC/MS	Early diagnosis	[105]
Idiopathic xerostomia	Caffeine	UPLC-QTOF- MS	Developing diagnostic markers and therapeutic strategies	[107]
pSS	Pyrimidine nucleotides, nucleosides	LC–MS	Biomarkers for diagnosis and treatment	[126]
M-TMD	Phenylacetate, dimethylamine, maltose, acetoin, isovalerate	^1^H-NMR	Diagnosing and monitoring treatment for musculoskeletal disorders	[109]
Apical root resorption	Butyrate, fumarate, α-linolenic acid	^1^H NMR	Early diagnosis and monitoring	[114]
WSLs	Proline, glycine	16S rRNA-seq UPLC-MS/MS	Biomarkers for the diagnosis and treatment of WSLs	[115]
Oral candidiasis	Tyrosine, choline, phosphoenolpyruvate	CE-TOF–MS	Verification of Candida presence	[110]
Halitosis	5-aminovaleric acid, *N*-acetylornithine	16S rRNA-seq	Identification of halitosis	[111]
RAU	Estrone sulfate, dehydroepiandrosterone sulfate	LC–MS/MS	Potential diagnostic utility	[112]
RIOM	Histidine, tyrosine	CE-TOF–MS	Predicting the severity of OM	[113]

### Oral precancerous lesions (OLP)

**Fig. 2:**
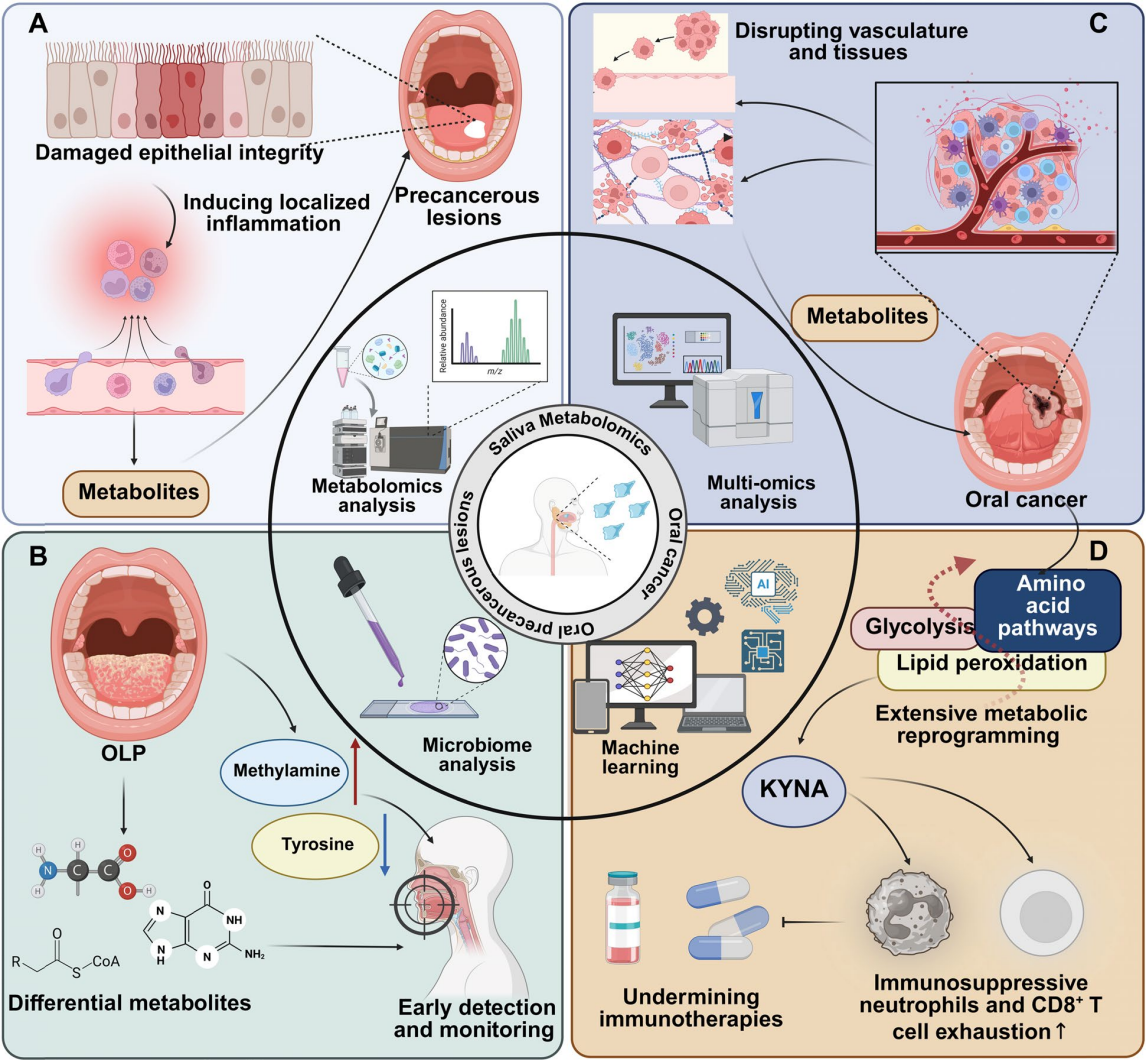
Salivary metabolomics in the diagnosis and management of oral precancerous lesion and oral cancer. **A** In oral precancerous lesions, epithelial damage disrupts barrier integrity, leading to the release of metabolic byproducts into saliva. **B** Microbiome and metabolomic analyses identify differential metabolites, enabling early detection and monitoring. **C** In oral cancer, disrupted tissue and vasculature facilitate the release of tumor-specific metabolites and inflammatory mediators into the oral cavity, resulting in a distinct biochemical profile detectable in saliva. **D** Saliva from OSCC patients, enriched with KYNA, promotes immunosuppressive neutrophil activity and induces CD8^+^ T cell exhaustion, compromising the efficacy of PD-L1 and IL-1β blockade therapies

Oral precancerous lesions subtly reshape the local oral environment, making saliva metabolites effective for early detection and monitoring [58]. These lesions alter epithelial integrity and induce localized inflammation, leading to the release of metabolic byproducts such as oxidative stress markers and inflammatory mediators into saliva [59] (Fig. 2A). The increased epithelial activity and metabolic remodeling in precancerous tissues result in the accumulation of disease-specific metabolites in saliva, capturing early pathological changes prior to systemic manifestation. For instance, salivary metabolomic profiling in patients with asymptomatic OLP reveals elevated levels of acetate, methylamine, and pyruvate, along with decreased tyrosine concentrations compared to healthy controls. Multivariate analysis identifies methylamine and tyrosine as potential biomarkers for distinguishing OLP. These findings suggest that salivary metabolites, particularly tyrosine, could serve as non-invasive biomarkers for early detection and monitoring of OLP progression [60]. Similarly, ultra-performance liquid chromatography coupled with high-resolution mass spectrometry identified 19 salivary metabolites differentiating OLP patients from healthy controls, including amino acid and lipid metabolites. A biomarker panel of three metabolites achieved diagnostic utility, revealing metabolic disruptions associated with OLP progression and highlighting potential pathways for early detection and understanding of its pathology [61]. Multi-omics analysis reveals significant shifts in the oral microbiome and metabolome of OLP patients compared to healthy controls. Increased abundance of specific bacterial genera such as *Pseudomonas* and distinct saliva metabolites correlates with OLP’s clinical features, suggesting microbial and metabolic components in its pathogenesis and potential for diagnostic biomarkers [62] (Fig. 2B). Additionally, salivary metabolomic profiling identifies key metabolites, including guanine, carnitine, and N-acetylputrescine, as potential biomarkers to differentiate oral leukoplakia from healthy controls, achieving high diagnostic accuracy. Additionally, 7-methylguanine distinguishes dysplastic from non-dysplastic OLP with moderate discrimination ability [63].

### Oral cancer

Oral cancer is characterized by profound metabolic reprogramming, distinguishing it from precancerous lesions and highlighting the value of saliva as a diagnostic medium in advanced disease [64]. Malignant tumors undergo extensive metabolic reprogramming, such as enhanced glycolysis, altered amino acid pathways, and lipid peroxidation, which result in a distinct biochemical profile detectable in saliva [65, 66, 67]. Furthermore, the invasive behavior of oral cancer disrupts vasculature and surrounding tissues, facilitating the release of tumor-specific metabolites and inflammatory mediators into the oral cavity. These cancer-driven alterations establish a unique saliva metabolic signature that closely mirrors disease progression and therapeutic response, providing a minimally invasive yet highly effective approach for monitoring malignancy [68]. Beyond serving as a diagnostic tool, saliva metabolites may actively contribute to oral cancer pathogenesis, saliva from oral squamous cell carcinoma (OSCC) patients, enriched in kynurenic acid (KYNA) and colonized by *Streptococcus mutans*, fosters OSCC progression in rat models. KYNA alters the tumor microenvironment, boosting immunosuppressive neutrophils and inducing CD8^+^ T cell exhaustion, undermining PD-L1 and IL-1β blockade therapies. These findings suggest targeting oral microbiota and metabolites could enhance OSCC treatments and prevention [69] (Fig. 2C, D). Additionally, saliva metabolomics reveals metabolic alterations in OSCC induced by dibenzo[def,p]pyrene (DB[a,l]P) in a mouse model. Untargeted LC–MS/MS profiling identifies significant enrichment of phosphatidic acid, a known mammalian target of rapamycin complex (mTORC) activator, and disruptions in lipid metabolism pathways [70]. Importantly, saliva metabolomics has shown great promise in the detection, prognosis prediction and treatment monitoring in oral cancer [71, 72]. For instance, saliva metabolomics in Japanese patients with OSCC identified 25 discriminatory metabolites, including choline, branched-chain amino acids, urea, and 3-hydroxybutyric acid. These findings highlight metabolic disruptions in OSCC and suggest potential biomarkers for non-invasive diagnosis [73]. Similarly, salivary metabolomic analysis identifies *N*-acetyl-d-glucosamine, l-pipecolic acid, and l-carnitine as key biomarkers for tongue squamous cell carcinoma, with a combination of these metabolites achieving an area under the curve (AUC) of 0.901 [74]. Additionally, metabolic profiling of OSCC identified key pathways, including the malate-aspartate shuttle, beta-alanine metabolism, and the Warburg effect. Salivary metabolites such as malic acid, maltose, protocatechuic acid, and catechol demonstrated high diagnostic potential [75]. Moreover, saliva metabolomics reveals distinct metabolite profiles in oral cavity squamous cell carcinoma (OCC), identifying glycine and proline as significantly altered in OCC compared to controls. Four metabolites, including glycine and proline, correlate with early-stage OCC. No significant metabolite differences were found in oral potentially malignant conditions (OPC) vs. controls or OCC with vs. without nodal metastasis. These findings highlight the potential of salivary metabolites as biomarkers for OCC detection and early-stage diagnosis [76]. An integrated LC/MS approach identified 14 salivary metabolites for the early diagnosis of OSCC. Five biomarkers—propionylcholine, N-acetyl-L-phenylalanine, sphinganine, phytosphingosine, and *S*-carboxymethyl-l-cysteine-yielded high diagnostic accuracy (AUC = 0.997), sensitivity (100%), and specificity (96.7%) in distinguishing early-stage OSCC from controls [77]. Likewise, salivary metabolomics revealed significant upregulation of metabolites such as 1-methylhistidine and sphinganine-1-phosphate in oral leukoplakia and OSCC, with downregulation of l-homocysteic acid and ubiquinone. These findings highlight the potential of salivary metabolites as biomarkers for early detection, malignant transformation prevention, and improved prognosis in oral cancer [78]. Conductive polymer spray ionization mass spectrometry (CPSI-MS) combined with machine learning distinguishes OSCC and premalignant lesions from healthy conditions with 86.7% accuracy. Dysregulated metabolites identified in saliva were validated at the tissue level using desorption electrospray ionization MS imaging, confirming altered metabolic pathways. Integrated metabolomic profiling of saliva and tumor tissues identified 17 overlapping metabolites differentiating oral cancer from controls, with two biomarkers achieving high diagnostic accuracy. This approach refines biomarker discovery by eliminating coincidental differences, advancing non-invasive screening for oral cancer [79]. Interestingly, salivary metabolite profiles correlated significantly with SUVmax in 18 F-fluorodeoxyglucose positron emission tomography/computed tomography (18 F-FDG PET/CT) among oral cancer patients, with 11 metabolites linked to delayed-phase maximum standardized uptake value (SUVmax). A logistic regression model utilizing two metabolites distinguished oral cancer patients from controls with an AUC of 0.738. These findings suggest salivary metabolites as potential non-invasive screening markers for identifying oral cancer patients with elevated SUVmax [80]. Notably, polyamine metabolites, including N1-acetylspermine (ASP), N8-acetylspermidine, and N1, N12-diacetylspermine, were quantitatively assessed using targeted metabolomics in the saliva and urine of head and neck cancer (HNC) patients. Elevated levels of ASP were detected in both saliva and urine from HNC patients compared to healthy controls. These results suggest that polyamine metabolites could serve as potential non-invasive biomarkers for the early diagnosis of HNC [81]. NMR spectroscopy identified significant salivary metabolic changes in head and neck squamous cell carcinoma, including elevated fucose and 1,2-propanediol and decreased proline levels. A biomarker combination of fucose, glycine, methanol, and proline achieved 92.1% classification accuracy, highlighting fucose as a potential diagnostic marker [82]. Alterations in saliva metabolite profiles are strongly associated with OSCC prognosis. Comprehensive salivary metabolomic analysis identified 3-methylhistidine as a significant prognostic factor for overall survival in OSCC. Elevated levels of 3-methylhistidine were associated with poorer outcomes, highlighting its potential as a non-invasive biomarker for predicting OSCC prognosis and guiding clinical management [83]. Importantly, profiling salivary metabolites effectively differentiates oral cancer from oral precancerous legions. For instance, salivary metabolomics identified indole-3-acetate and ethanolamine phosphate as key markers distinguishing OSCC from OLP, achieving high diagnostic accuracy [84]. Similarly, metabolic profiling using GC–MS identified 15 salivary metabolites, including decanedioic acid, l-proline, and pentanoic acid, that differ significantly among OSCC, OLK, and healthy controls. These metabolites demonstrate potential as tumor-specific biomarkers for early diagnosis and prediction of OSCC and OLK [85].

### Periodontal diseases

**Fig. 3:**
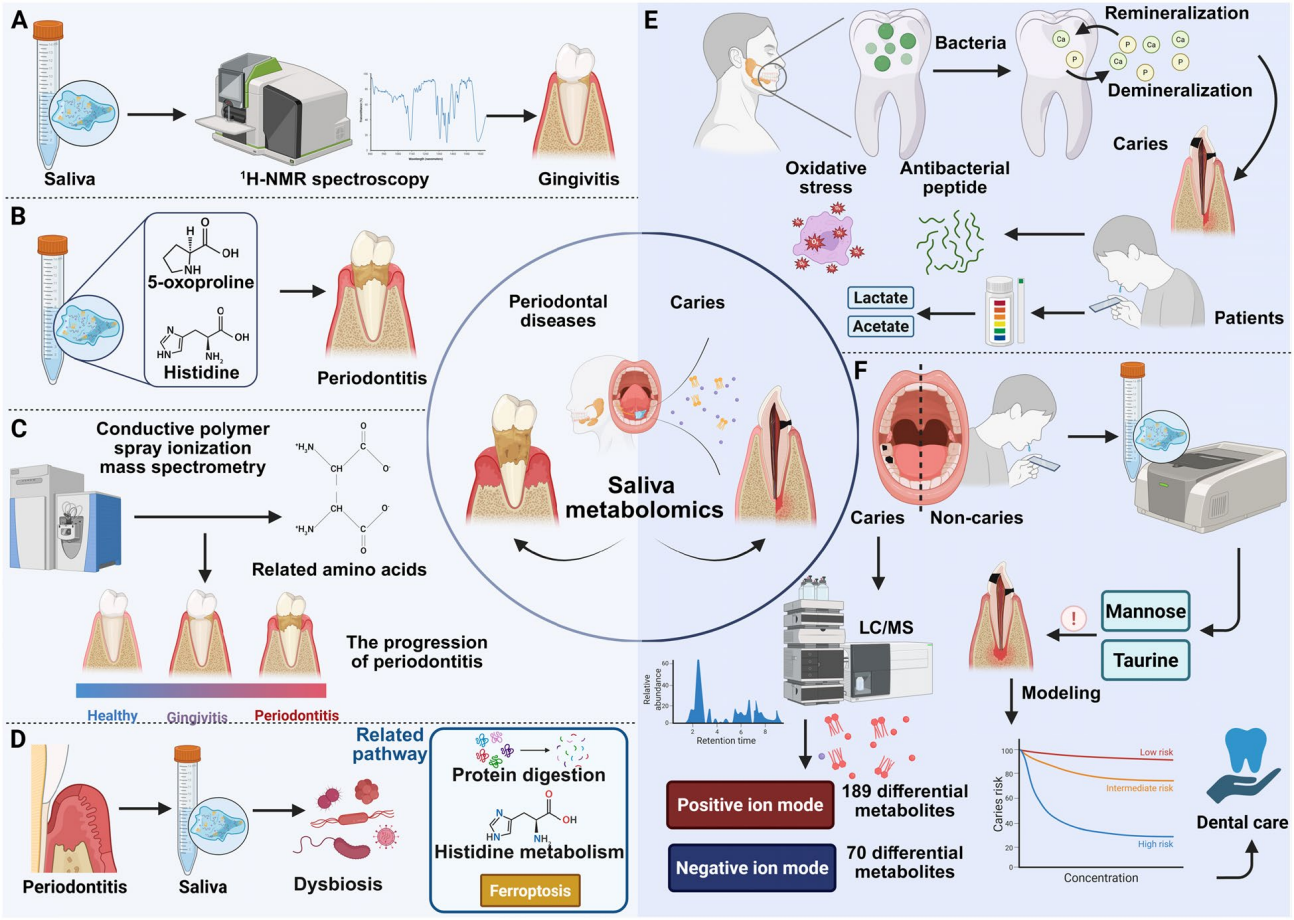
Saliva metabolomics in the diagnosis and monitoring of periodontal and caries diseases.** A** ^1^H-NMR spectroscopy is used to analyze saliva samples for the identification of biomarkers associated with gingivitis. **B** Salivary metabolites, such as 5-oxoproline and histidine can serve as key biomarkers reflecting the severity of periodontal inflammation. **C** Analysis of saliva metabolites using conductive polymer spray ionization mass spectrometry, emphasizing the critical role of amino acid metabolism in the progression of periodontitis. **D** In periodontitis patients, significant changes in the microbial composition of saliva have been observed, particularly in pathways related to protein digestion, histidine metabolism, and ferroptosis, compared to healthy controls. **E** The metabolic activity of cariogenic bacteria generates acid-producing byproducts like lactate and acetate, which can be easily detected in saliva. **F** The presentation of differences in saliva metabolites in positive and negative ion modes, with key metabolites such as taurine and mannose, shows great potential as reliable biomarkers for caries

Periodontal diseases lead to significant metabolic changes in the oral environment, driven by microbial imbalance, inflammation, and tissue destruction. These alterations are mirrored in saliva, which directly reflects the condition of the periodontal tissues. Inflammatory markers, proteases, and lipid peroxidation products released during disease progression can be detected in saliva, making it a valuable medium for monitoring periodontal disease. Saliva metabolomics offers the potential to identify biomarkers linked to microbial shifts and host immune responses, facilitating early detection, real-time monitoring, and evaluation of treatment efficacy in periodontal diseases. For instance, salivary metabolite profiling using ^1^H-NMR spectroscopy reveals distinct metabolic signatures associated with early gingival inflammation, as indicated by the full-mouth bleeding score. Specific metabolites linked to enzymatic activities of oral bacteria may serve as indicators of preclinical gingivitis, distinguishing individuals with early inflammatory changes. This approach suggests the potential for salivary biomarkers to detect gingivitis at an early, subclinical stage [86] (Fig. 3A). Likewise, salivary metabolomics identified cadaverine, 5-oxoproline, and histidine as key biomarkers reflecting periodontal inflammation severity, with high diagnostic accuracy. Post-debridement samples improved detection of subgingival metabolites, providing a refined model for monitoring periodontitis activity [87] (Fig. 3B).

In addition, profiling saliva metabolites using conductive polymer spray ionization mass spectrometry provides a rapid, non-invasive method for detecting severe periodontitis. Dysregulated metabolites, particularly amino acids, were identified in patients with severe periodontitis, with notable changes observed after plaque removal. These findings underscore amino acid metabolism as a key player in periodontitis progression [88] (Fig. 3C). Moreover, untargeted salivary metabolomics identified ethanol, taurine, isovalerate, butyrate, and glucose as robust biomarkers distinguishing periodontitis from healthy controls. A metabolite panel demonstrated high diagnostic accuracy across cohorts, offering potential for periodontal screening and therapy monitoring [89]. Similarly, salivary metabolomics distinguished chronic and aggressive periodontitis (AgP) patients from healthy individuals with 81% accuracy, identifying altered levels of pyruvate, lactate, proline, phenylalanine, and tyrosine. However, metabolic profiles of chronic and AgP overlapped, highlighting shared pathways in disease mechanisms [90]. Notably, salivary metabolomics revealed age-dependent associations with periodontal variables, with phenylacetate consistently linked to periodontal tissue destruction and bacterial activity across all age groups. These findings highlight phenylacetate as a promising biomarker for periodontitis and provide insights into host-microbe metabolic interactions in oral health [91]. Interestingly, salivary metabolite profiling identified 84 metabolites linked to periodontal health, with 2-pyrrolidineacetic acid and butyrylputrescine emerging as consistent markers of oral dysbiosis. Nine baseline metabolites were associated with future tooth loss, reflecting processes such as tissue destruction and cell proliferation [92]. Saliva metabolomics reveals distinct metabolic signatures linked to periodontitis and systemic diseases, particularly in type 2 diabetes. Metabolites such as threonate, cadaverine, and lactate correlate with periodontal inflammation, while disruptions in liver lipid metabolism are associated with increased cardiometabolic risk. These findings underscore the potential of saliva as a non-invasive diagnostic tool for assessing both oral and systemic health [93]. Therapeutic interventions can significantly alter the salivary metabolomic profile in periodontal disease, but full alignment with a healthy state is not observed. Non-surgical periodontal therapy (NST) significantly altered the salivary metabolomic profile in generalized periodontitis patients, with partial least squares (PLS) achieving 100% accuracy in distinguishing pre- and post-treatment profiles. Despite improved clinical parameters, post-NST profiles remained distinct from healthy individuals, highlighting persistent metabolic differences associated with periodontal disease. Similarly, NST improved clinical parameters in generalized chronic periodontitis but did not fully restore the salivary metabolomic profile to that of healthy controls. NMR spectroscopy revealed distinct metabolic changes post-treatment, with differences in metabolites such as lactate, pyruvate, and tyrosine persisting between treated and healthy individuals, underscoring a retained metabolic signature of disease [21]. Integrating oral microbiome and salivary metabolite profiling enhances the understanding of periodontal disease pathogenesis. For instance, salivary microbiome and metabolome analysis in periodontitis patients reveals significant changes in microbial composition and 103 differential metabolites compared to healthy controls. Dysbiosis is associated with upregulated pathways in protein digestion, histidine metabolism, and ferroptosis, while tryptophan and glutathione metabolism are altered in patients. Correlation analysis links clinical parameters with pathogen abundance and disease-related metabolites, suggesting that salivary profiling could serve as a valuable tool for diagnosing, monitoring, and understanding periodontitis pathogenesis [94] (Fig. 3D). Similarly, saliva metabolomics and microbiome profiling reveal distinct microbial and metabolic signatures in generalized periodontitis. Elevated levels of *Treponema*, *Peptococcus*, and other genera, alongside metabolites such as urea, beta-alanine, and thymine, were associated with aggressive and chronic periodontitis. Key pathways, including pyrimidine and arginine metabolism, were significantly altered between disease subtypes [95].

### Caries

Caries is uniquely suited for saliva metabolite-based biomarker discovery due to the direct interaction between saliva and tooth surfaces, where early demineralization occurs [96]. The metabolic activity of cariogenic bacteria, such as *Streptococcus mutans*, produces acidogenic byproducts like lactate and acetate, which are readily detectable in saliva [97]. Furthermore, the dynamic equilibrium between demineralization and remineralization processes in the enamel is reflected in changes to salivary calcium and phosphate levels [98] (Fig. 3E). Saliva also captures host responses, including shifts in oxidative stress markers and antimicrobial peptides, which modulate the cariogenic environment. These localized biochemical changes provide a sensitive and non-invasive means of detecting caries at its earliest stages, monitoring disease progression, and evaluating therapeutic interventions. For instance, salivary metabolomics identified distinct profiles in children with active carious lesions compared to healthy controls, highlighting differences in saccharides and amino acids, including elevated taurine and mannose in caries cases. Predictive modeling using these metabolites achieved moderate accuracy, suggesting their potential as biomarkers for caries risk assessment. These findings underscore the value of salivary metabolomics in advancing personalized approaches to dental caries prevention and management [99]. Notably, saliva metabolomics reveals distinct metabolic profiles between caries-active and caries-free children, with 189 differential metabolites identified in the positive ion mode and 70 in the negative ion mode. Key metabolites, including histamine, L-histidine, and succinate, were enriched in the caries-active group and linked to altered metabolic pathways [100] (Fig. 3F). Additionally, altered salivary metabolomes and microbiomes are linked to severe early childhood caries (SECC). Key metabolites, including 2-benzylmalate, epinephrine, and 3-Indoleacrylic acid, correlate with caries status, revealing disrupted pathways such as amino acid, purine, and pyrimidine metabolism. Specific oral bacteria, including *Veillonella* and *Porphyromonas*, are associated with differential metabolites. These findings suggest that salivary metabolites and microbial profiles may serve as biomarkers for SECC, offering insights into its pathophysiology and potential avenues for personalized prevention and treatment strategies [101].

### Burning mouth syndrome

Burning mouth syndrome (BMS) is a chronic pain disorder marked by persistent burning sensations in the oral cavity, typically in the absence of observable clinical lesions, thereby underscoring the need for reliable non-invasive diagnostic approaches [102]. Saliva directly interacts with affected oral tissues and reflects localized neurogenic inflammation, oxidative stress, and altered epithelial metabolism, all of which are implicated in BMS pathophysiology. Metabolomic changes in saliva provide unique insights into the underlying mechanisms of pain and sensory dysfunction. For instance, in patients with BMS, particularly those exhibiting psychiatric symptoms such as depression or anxiety, analyses of oral microbiota and salivary metabolites reveal no significant microbial differences from healthy controls, but a reduction in microbial diversity and an increase in pro-inflammatory species. Metabolomic profiling highlights alterations in amino acid and lipid metabolism linked to immunological responses, suggesting these factors may influence the pathogenesis and management of BMS [103]. Additionally, salivary metabolomics in BMS revealed 394 differentially expressed metabolites, with significant downregulation of the caffeine metabolism pathway and reduced levels of caffeine and its metabolites, paraxanthine and theophylline. Pathway enrichment analysis identified 30 key metabolites linked to 25 metabolic pathways [104]. Moreover, a comparative analysis of the salivary metabolome in primary BMS revealed shifts in tyrosine metabolism, including l-dopa, l-tyrosine, and tyramine, identified using LC/MS. These changes may indicate an adaptive response to chronic pain or impaired dopaminergic transmission. However, no significant differences in cytokines, neuroinflammatory markers, or steroid hormones were detected, emphasizing the role of metabolomic alterations over inflammatory pathways in BMS pathophysiology [105].

### Xerostomia

Xerostomia, or dry mouth, results from altered salivary gland function, leading to significant shifts in the composition of saliva [106]. The altered saliva metabolite profile provides direct insights into glandular dysfunction and its downstream effects on oral health. Additionally, saliva reflects local immune responses and compensatory metabolic adaptations, offering a comprehensive profile of xerostomia's pathophysiology. Salivary metabolomic profiling identifies 195 differentially expressed metabolites in idiopathic xerostomia, with alterations notably concentrated in the caffeine metabolism pathway. This pathway's disruption underscores a potential neuropathic influence in the disease's pathology. These insights offer a foundation for developing diagnostic markers and therapeutic strategies for xerostomia [107]. Additionally, metabolomic profiling of saliva revealed distinct metabolic signatures in dry mouth conditions associated with head and neck cancer (HNC) and primary Sjögren's syndrome (pSS). Both groups exhibited elevated salivary pyrimidine nucleotides and nucleosides, implicating purinergic signaling, alongside dysregulated amino acid metabolism. Notably, DL-3-aminoisobutyric acid levels were significantly higher in HNC patients, with a similar trend in pSS. These findings enhance understanding of dry mouth pathophysiology and suggest potential biomarkers for diagnosis and treatment [108].

### Temporomandibular disorders

Saliva captures metabolites and inflammatory mediators associated with muscle metabolism and dysfunction, reflecting biochemical changes linked to TMD of muscular origin (M-TMD). For instance, salivary metabolomic profiling of patients with TMDs of muscular origin reveals eight key metabolites, including l-isoleucine, methylmalonic acid, and lactic acid, distinguishing them from healthy controls. NMR-based metabolomics, coupled with multivariate analysis, provides valuable insights into the metabolic alterations associated with TMDs, highlighting its potential for understanding the pathogenesis and identifying biomarkers for this condition. Similarly, salivary metabolomics in women with TMD of muscular origin reveals distinct metabolic profiles characterized by changes in phenylacetate, dimethylamine, maltose, acetoin, and isovalerate. Post-treatment profiles show reduced distinctions from controls, indicating a shift toward normalization. These metabolites emerge as potential biomarkers for TMD, highlighting the utility of salivary analysis in diagnosing and monitoring treatment outcomes for musculoskeletal disorders [109].

### Other oral diseases

Saliva metabolomics provides a comprehensive approach to understanding the biochemical changes underlying diverse oral diseases, revealing disease-specific metabolic signatures. For instance, salivary metabolomics reveals distinct metabolic alterations associated with oral candidiasis. Analysis identified 51 metabolites, with significant changes in both unstimulated and stimulated saliva of Candida-positive individuals. Elevated levels of tyrosine, choline, and phosphoenolpyruvate, among others, were observed in unstimulated saliva, while metabolites like ornithine and butyrate were decreased in stimulated saliva [110]. Additionally, halitosis was associated with distinct salivary microbiota and metabolite profiles. Elevated levels of *Prevotella*, *Alloprevotella*, and *Megasphaera*, alongside increased 5-aminovaleric acid and *N*-acetylornithine, characterized the halitosis group. Correlations suggest *Alloprevotella* and *Prevotella* contribute to cadaverine and putrescine pathways, shedding light on halitosis mechanisms [111]. Notably, saliva from recurrent aphthous ulcer (RAU) patients showed significant metabolic alterations, including decreased estrone sulfate and dehydroepiandrosterone sulfate, implicating imbalances in tryptophan metabolism and steroid hormone biosynthesis. These findings suggest metabolic disruptions linked to psychogenic, endocrine, and immunological factors in RAU, with potential diagnostic utility for identified metabolites [112]. Radiotherapy-induced oral mucositis (RIOM) in HNC patients was linked to distinct salivary metabolites. Pretreatment levels of histidine and tyrosine significantly differentiated high-grade from low-grade mucositis, while gamma-aminobutyric acid and 2-aminobutyric acid were elevated in severe cases. These findings suggest salivary metabolomic profiling as a tool for predicting mucositis severity and guiding timely interventions [113].

### Orthodontic therapy-related complications

**Fig. 4:**
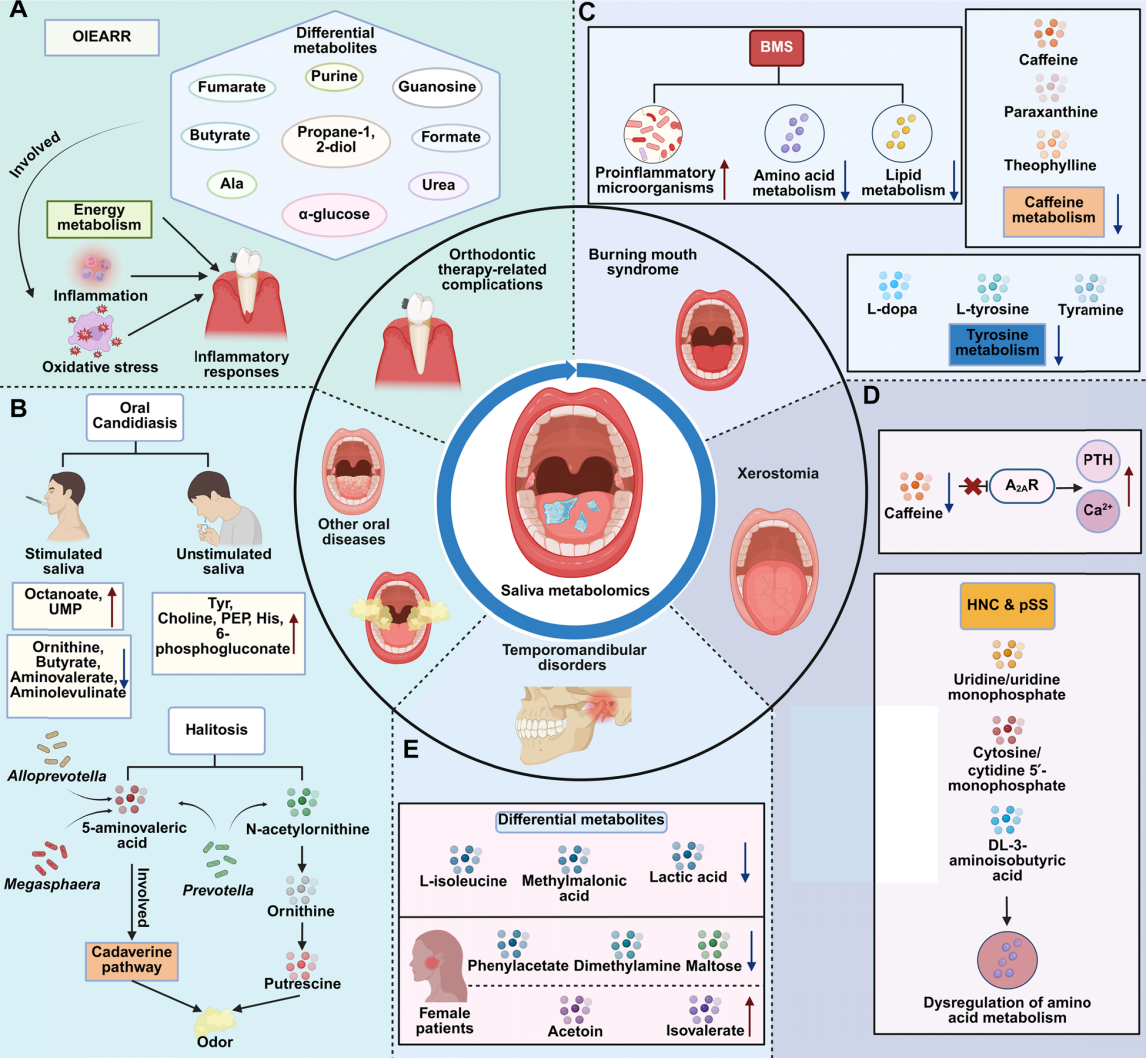
Saliva metabolomics in the diagnosis and monitoring of various oral diseases.** A** OIEARR shows changes in salivary metabolites such as butyrate, fumarate, and α-linolenic acid, reflecting inflammation, oxidative stress, and dysregulation of energy metabolism in periodontal tissues. **B** Oral candidiasis presents distinct metabolite profiles in both unstimulated and stimulated saliva. In halitosis, elevated levels of 5-aminovaleric acid and *N*-acetylornithine, along with microbial contributions, are observed. **C** In BMS, disruptions in amino acid and lipid metabolism are linked to immune responses. Caffeine metabolism is downregulated, and alterations in tyrosine metabolism may contribute to mouth dryness in xerostomia. **D** In xerostomia, elevated pyrimidine nucleotides and DL-3-aminoisobutyric acid, along with disrupted amino acid metabolism, are observed. **E** TMDs are associated with metabolites including L-isoleucine, methylmalonic acid, and lactic acid, whereas inn women with TMDs, distinct metabolic profiles are observed, characterized by alterations in phenylacetate, dimethylamine, maltose, acetoin, and isovalerate

Saliva metabolomics provides critical insights into the biochemical and microbial changes induced by orthodontic treatments, uncovering associations with therapy-related complications. For instance, orthodontically induced external apical root resorption (OIEARR) is associated with altered salivary metabolites, including butyrate, fumarate, and α-linolenic acid, reflecting inflammation, oxidative stress, and energy metabolism dysregulation in periodontal tissues. Salivary metabolomics using ^1^H NMR demonstrated effective discrimination between patients with and without OIEARR, highlighting its potential for early diagnosis and monitoring [114]. Interestingly, adolescents undergoing clear aligner therapy are at increased risk for developing white spot lesions (WSLs), potentially due to alterations in the oral microbiome and metabolome. Analysis revealed higher abundances of specific taxa, including *Lachnoanaerobaculum*, *Rothia*, and *Subdoligranulum*, in those with WSLs. Metabolomic changes, particularly in amino acids like proline and glycine, were associated with disrupted metabolic pathways. These findings highlight the role of oral microbiota dysbiosis in WSL development, offering potential biomarkers for early detection and management of WSLs linked to clear aligner use [115] (Fig. 4).

Collectively, these findings underscore the clinical relevance of salivary metabolomics across a wide range of oral diseases. Salivary metabolomics has demonstrated substantial promise across the clinical spectrum of oral diseases, with applications in early diagnosis, prognostic stratification, and longitudinal disease monitoring. From precancerous lesions and OSCC to periodontitis, caries, and therapy-associated complications, salivary metabolite profiles have consistently distinguished disease states from health, reflected disease severity, and dynamically responded to therapeutic interventions [60, 69, 87, 98]. Importantly, longitudinal studies have shown that these profiles evolve in parallel with clinical progression or remission, indicating their potential as non-invasive biomarkers to track the natural course of disease and evaluate treatment outcomes in real time. However, its clinical translation requires careful consideration of biological variability—including age, circadian rhythm, diet, and oral microbiota—as well as technical factors related to sample handling and analytical reproducibility. Integrating salivary metabolomics with clinical, microbiological, and imaging data through multi-modal approaches may further enhance diagnostic precision and biological interpretability [50, 51, 53]. With continued validation in large, prospective cohorts and the establishment of standardized workflows, salivary metabolomics holds considerable potential to support personalized, longitudinal management of oral diseases.

## Challenges and perspectives

Despite its considerable promise as a non-invasive diagnostic tool, the clinical application of saliva metabolomics faces several distinct challenges. These stem from the inherent complexity of saliva as a biological fluid and the dynamic, multifactorial nature of the metabolic processes it represents [116].

Firstly, one significant challenge in applying saliva metabolomics is the difficulty in establishing clear, direct links between specific metabolomic changes in saliva and the underlying mechanisms for oral diseases. Unlike blood, which is a more stable biofluid and reflects systemic changes more directly, saliva is influenced by both local oral conditions and systemic factors. The dynamic interplay between the oral microbiome, host immune responses, and environmental factors complicates the interpretation of metabolic changes. In diseases such as oral cancer or periodontal disease, salivary metabolite profiles may overlap with systemic diseases like diabetes or cardiovascular disorders, complicating efforts to distinguish disease-specific biomarkers. This complexity underscores the need for a deeper understanding of how salivary metabolic alterations specifically reflect disease mechanisms [117].

**Fig. 5:**
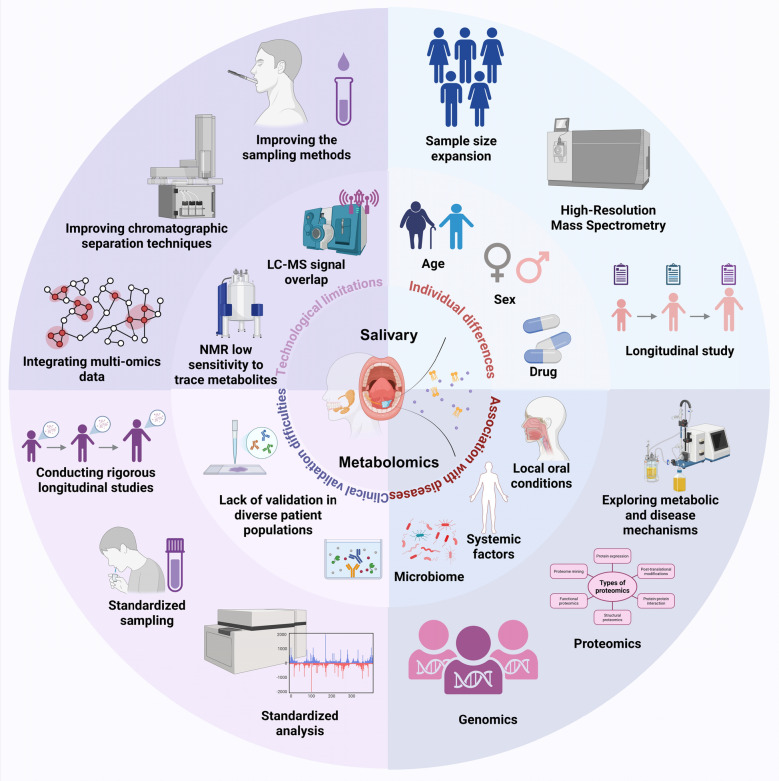
Challenges and prospects in salivary metabolomics. In the technological aspect, it shows challenges like LC–MS signal overlap, low NMR sensitivity, and the complexity of salivary composition, while suggesting solutions such as improved chromatographic techniques, integration of multi-omics data, and enhanced sampling methods. In terms of clinical application, it points out the need for validation in diverse populations and the issue of inconsistent analysis methods, with strategies including standardized sampling, standardized data analysis, and rigorous longitudinal studies to boost reliability. Individual differences also play a role, as age, sex, medication, microbiome, and systemic conditions can affect salivary composition. Expanding sample sizes, longitudinal studies, and accounting for individual differences can improve the specificity of biomarkers. Finally, the complex interplay of local oral conditions, systemic factors, and disease mechanisms is depicted, indicating that a multi-disciplinary approach combining metabolomics, proteomics, and genomics is essential for exploring and validating disease-specific biomarkers

Secondly, saliva composition is highly individualized, influenced by a range of factors such as age, sex, diet, oral hygiene, genetics, and medication use. For instance, systemic antibiotic use in the past three months was strongly linked to elevated levels of taurine, glycine, and ornithine. These findings highlight the influence of external factors such as antibiotics on salivary metabolites [118] (Fig. 5C). Similarly, salivary metabolite profiles for oral cancer detection are influenced by collection timing post-meal. Metabolomic analysis identified 51 discriminatory metabolites at 12 h post-dinner, compared to fewer at 1.5 and 3.5 h after breakfast. The 12-h fasting period yielded the highest diagnostic accuracy, emphasizing its importance for standardized saliva collection in biomarker discovery. This inherent variability makes it challenging to establish consistent diagnostic biomarkers across different individuals [119]. Additionally, the oral microbiome and salivary gland function vary widely between individuals, and these factors can significantly alter the metabolic profile of saliva. To address this variability, improving the precision of metabolomics techniques through better analytical methods, such as high-resolution mass spectrometry and more robust data normalization procedures, could help reduce confounding effects (Fig. 5A). Furthermore, expanding clinical cohorts to include diverse populations and accounting for these external factors in study design could increase the reliability of findings. Longitudinal studies that track individual baseline profiles, accounting for diet, medication, and health status, may also help to differentiate disease-specific biomarkers from those influenced by external variables. These strategies will enhance the clinical applicability of salivary metabolomics by providing more standardized and reliable biomarkers across different patient groups [120] (Fig. 5D).

Thirdly, despite significant advances in analytical techniques such as LC–MS, GC–MS, and NMR spectroscopy, there remain technical limitations that hinder the precision and comprehensiveness of salivary metabolomic analyses. For instance, while LC–MS provides high sensitivity for detecting metabolites, the complexity of saliva, which contains a wide range of metabolites, proteins, and microbial products, can lead to challenges in resolving overlapping signals and distinguishing low-abundance metabolites [121]. Furthermore, NMR, although capable of providing structural information, lacks the sensitivity and resolution of mass spectrometry for detecting metabolites at low concentrations. These limitations can lead to incomplete or inaccurate metabolic profiling, which impedes the identification of robust biomarkers for clinical use. To address these challenges, strategies such as improved sample preparation, enhanced chromatographic techniques, and the use of hyperpolarization in NMR can enhance sensitivity and resolution [122, 123]. Additionally, integrating multi-omics data and employing advanced bioinformatics tools can refine data analysis, reduce variability, and improve the reliability of findings, ultimately increasing the clinical applicability of salivary metabolomics [124].

Lastly, the clinical validation of salivary biomarkers represents a significant challenge. While preliminary studies highlight the potential of saliva metabolomics in identifying disease-specific biomarkers, much of the research remains in the discovery phase. To achieve clinical applicability, these biomarkers must undergo rigorous validation across heterogeneous patient populations [125]. This requires well-designed longitudinal studies to establish the reliability and reproducibility of biomarkers in early disease detection, monitoring disease progression, and evaluating therapeutic responses. In the absence of robust validation, the integration of saliva metabolomics into clinical practice remains limited (Fig. 5B).

## Conclusion

In summary, salivary metabolomics represents a promising non-invasive approach for the diagnosis and monitoring of a broad spectrum of oral diseases. Its capacity to reflect both localized and systemic metabolic alterations offers distinct advantages for early detection, disease progression tracking, and assessment of therapeutic efficacy. However, several challenges remain, including incomplete understanding of the mechanistic links between salivary metabolites and disease processes, substantial inter-individual variability, and the complex dynamics of the oral microenvironment. Furthermore, technical limitations, data interpretation complexities, and the lack of large-scale clinical validation hinder its routine clinical implementation. Despite these obstacles, continued advancements in analytical technologies, standardization protocols, and multi-omics integration are expected to enhance the diagnostic accuracy and clinical relevance of salivary metabolomics. Ultimately, this approach holds the potential to transform the landscape of oral disease management through more personalized, precise, and mechanism-informed strategies.

## Data Availability

The data generated are included within the manuscript.

## References

[1] Ziober BL, Mauk MG, Falls EM, Chen Z, Ziober AF, Bau HH. Lab-on-a-chip for oral cancer screening and diagnosis. Head Neck. 2008;30(1):111–21. 17902150 10.1002/hed.20680

[2] Zimmermann BG, Park NJ, Wong DT. Genomic targets in saliva. Ann N Y Acad Sci. 2007;1098:184–91. 17435127 10.1196/annals.1384.002 PMC2910758

[3] Hyvarinen E, Savolainen M, Mikkonen JJW, Kullaa AM. Salivary metabolomics for diagnosis and monitoring diseases: challenges and possibilities. Metabolites. 2021;11(9):587. 34564402 10.3390/metabo11090587 PMC8469343

[4] Spielmann N, Wong DT. Saliva: diagnostics and therapeutic perspectives. Oral Dis. 2011;17(4):345–54. 21122035 10.1111/j.1601-0825.2010.01773.x PMC3056919

[5] Cui L, Liu J, Yan X, Hu S. Identification of metabolite biomarkers for gout using capillary ion chromatography with mass spectrometry. Anal Chem. 2017;89(21):11737–43. 28972752 10.1021/acs.analchem.7b03232

[6] Hu S, Xie Y, Ramachandran P, Ogorzalek Loo RR, Li Y, Loo JA, Wong DT. Large-scale identification of proteins in human salivary proteome by liquid chromatography/mass spectrometry and two-dimensional gel electrophoresis-mass spectrometry. Proteomics. 2005;5(6):1714–28. 15800970 10.1002/pmic.200401037

[7] Lim PW, Garssen J, Sandalova E. Potential use of salivary markers for longitudinal monitoring of inflammatory immune responses to vaccination. Mediators Inflamm. 2016;2016:6958293. 27022211 10.1155/2016/6958293 PMC4789015

[8] Kumar P, Gupta S, Das BC. Saliva as a potential non-invasive liquid biopsy for early and easy diagnosis/prognosis of head and neck cancer. Transl Oncol. 2024;40: 101827. 38042138 10.1016/j.tranon.2023.101827 PMC10701368

[9] Li Y, Ou Y, Fan K, Liu G. Salivary diagnostics: opportunities and challenges. Theranostics. 2024;14(18):6969–90. 39629130 10.7150/thno.100600 PMC11610148

[10] Papale F, Santonocito S, Polizzi A, Giudice AL, Capodiferro S, Favia G, Isola G. The new era of salivaomics in dentistry: frontiers and facts in the early diagnosis and prevention of oral diseases and cancer. Metabolites. 2022;12(7):638. 35888762 10.3390/metabo12070638 PMC9319392

[11] Belstrom D. The salivary microbiota in health and disease. J Oral Microbiol. 2020;12(1):1723975. 32128039 10.1080/20002297.2020.1723975 PMC7034443

[12] Garcia PN, de Souza MM, Izidoro MA, Juliano L, Lourenço SV, Camillo CMC. Saliva metabolomics: concepts and applications in oral disorders. Clin Oral Investig. 2024;28(11):579. 39377832 10.1007/s00784-024-05990-y

[13] Kapel-Regula A, Dus-Ilnicka I, Radwan-Oczko M. Relevance of saliva analyses in terms of etiological factors, biomarkers, and indicators of disease course in patients with multiple sclerosis—a review. Int J Mol Sci. 2024;25(23):12559. 39684271 10.3390/ijms252312559 PMC11641500

[14] Kinane DF, Gabert J, Xynopoulos G, Guzeldemir-Akcakanat E. Strategic approaches in oral squamous cell carcinoma diagnostics using liquid biopsy. Periodontol 2000. 2024;96(1):316–28. 38676371 10.1111/prd.12567 PMC11579816

[15] Aihara M, Jinnouchi H, Yoshida A, Ijima H, Sakurai Y, Hayashi T, Koizumi C, Kubota T, Usami S, Yamauchi T, Sakata T, Kadowaki T, Kubota N. Evaluation of glycated albumin levels in tears and saliva as a marker in patients with diabetes mellitus. Diabetes Res Clin Pract. 2023;199: 110637. 36963507 10.1016/j.diabres.2023.110637

[16] Abdul Rehman S, Khurshid Z, Hussain Niazi F, Naseem M, Al Waddani H, Sahibzada HA, Sannam KR. Role of salivary biomarkers in detection of cardiovascular diseases (CVD). Proteomes. 2017;5(3):21. 28783097 10.3390/proteomes5030021 PMC5620538

[17] Adamashvili I, Pressly T, Gebel H, Milford E, Wolf R, Mancini M, Sittg K, Ghali GE, Hall V, McDonald JC. Soluble HLA in saliva of patients with autoimmune rheumatic diseases. Rheumatol Int. 2002;22(2):71–6. 12070679 10.1007/s00296-002-0173-3

[18] Umapathy VR, Natarajan PM, Swamikannu B. Molecular and therapeutic roles of non-coding rnas in oral cancer—a review. Molecules. 2024;29(10):2402. 38792263 10.3390/molecules29102402 PMC11123887

[19] Ebersole JL, Hasturk H, Huber M, Gellibolian R, Markaryan A, Zhang XD, Miller CS. Realizing the clinical utility of saliva for monitoring oral diseases. Periodontol 2000. 2024;95(1):203–19. 39010260 10.1111/prd.12581

[20] Bastias D, Maturana A, Marin C, Martinez R, Niklander SE. Salivary biomarkers for oral cancer detection: an exploratory systematic review. Int J Mol Sci. 2024;25(5):2634. 38473882 10.3390/ijms25052634 PMC10932009

[21] Citterio F, Romano F, Meoni G, Iaderosa G, Grossi S, Sobrero A, Dego F, Corana M, Berta GN, Tenori L, Aimetti M. Changes in the salivary metabolic profile of generalized periodontitis patients after non-surgical periodontal therapy: a metabolomic analysis using nuclear magnetic resonance spectroscopy. J Clin Med. 2020;9(12):3977. 33302593 10.3390/jcm9123977 PMC7763572

[22] Liu J, Huang D, Cai Y, Cao Z, Liu Z, Zhang S, Zhao L, Wang X, Wang Y, Huang F, Wu Z. Saliva diagnostics: emerging techniques and biomarkers for salivaomics in cancer detection. Expert Rev Mol Diagn. 2022;22(12):1077–97. 36631426 10.1080/14737159.2022.2167556

[23] Pfaffe T, Cooper-White J, Beyerlein P, Kostner K, Punyadeera C. Diagnostic potential of saliva: current state and future applications. Clin Chem. 2011;57(5):675–87. 21383043 10.1373/clinchem.2010.153767

[24] Tomita A, Mori M, Hiwatari K, Yamaguchi E, Itoi T, Sunamura M, Soga T, Tomita M, Sugimoto M. Effect of storage conditions on salivary polyamines quantified via liquid chromatography–mass spectrometry. Sci Rep. 2018;8(1):12075. 30104641 10.1038/s41598-018-30482-x PMC6089938

[25] Duarte D, Castro B, Pereira JL, Marques JF, Costa AL, Gil AM. Evaluation of saliva stability for NMR metabolomics: collection and handling protocols. Metabolites. 2020;10(12):515. 33352779 10.3390/metabo10120515 PMC7766053

[26] Zhong L, Cheng F, Lu X, Duan Y, Wang X. Untargeted saliva metabonomics study of breast cancer based on ultra performance liquid chromatography coupled to mass spectrometry with HILIC and RPLC separations. Talanta. 2016;158:351–60. 27343615 10.1016/j.talanta.2016.04.049

[27] Figueira J, Gouveia-Figueira S, Öhman C, Lif Holgerson P, Nording ML, Öhman A. Metabolite quantification by NMR and LC-MS/MS reveals differences between unstimulated, stimulated, and pure parotid saliva. J Pharm Biomed Anal. 2017;140:295–300. 28380387 10.1016/j.jpba.2017.03.037

[28] Fu J, Zhu F, Xu CJ, Li Y. Metabolomics meets systems immunology. EMBO Rep. 2023;24(4): e55747. 36916532 10.15252/embr.202255747 PMC10074123

[29] Danzi F, Pacchiana R, Mafficini A, Scupoli MT, Scarpa A, Donadelli M, Fiore A. To metabolomics and beyond: a technological portfolio to investigate cancer metabolism. Signal Transduct Target Ther. 2023;8(1):137. 36949046 10.1038/s41392-023-01380-0 PMC10033890

[30] Alarcon-Barrera JC, Kostidis S, Ondo-Mendez A, Giera M. Recent advances in metabolomics analysis for early drug development. Drug Discov Today. 2022;27(6):1763–73. 35218927 10.1016/j.drudis.2022.02.018

[31] Segers K, Declerck S, Mangelings D, Heyden YV, Eeckhaut AV. Analytical techniques for metabolomic studies: a review. Bioanalysis. 2019;11(24):2297–318. 31845604 10.4155/bio-2019-0014

[32] Collins SL, Koo I, Peters JM, Smith PB, Patterson AD. Current challenges and recent developments in mass spectrometry-based metabolomics. Annu Rev Anal Chem (Palo Alto Calif). 2021;14(1):467–87. 34314226 10.1146/annurev-anchem-091620-015205

[33] Lella C, Nestor L, De Bundel D, Vander Heyden Y, Van Eeckhaut A. Targeted chiral metabolomics of d-amino acids: their emerging role as potential biomarkers in neurological diseases with a focus on their liquid chromatography–mass spectrometry analysis upon chiral derivatization. Int J Mol Sci. 2024;25(22):12410. 39596475 10.3390/ijms252212410 PMC11595108

[34] Mandal V, Ajabiya J, Khan N, Tekade RK, Sengupta P. Advances and challenges in non-targeted analysis: an insight into sample preparation and detection by liquid chromatography-mass spectrometry. J Chromatogr A. 2024;1737: 465459. 39476774 10.1016/j.chroma.2024.465459

[35] Sarkar J, Singh R, Chandel S. Understanding LC/MS-based metabolomics: a detailed reference for natural product analysis. Proteomics Clin Appl. 2024;2024: e202400048. 10.1002/prca.202400048 39474988

[36] Liu J, Zhao H, Yin Z, Dong H, Chu X, Meng X, Li Y, Ding X. Application and prospect of metabolomics-related technologies in food inspection. Food Res Int. 2023;171: 113071. 37330829 10.1016/j.foodres.2023.113071

[37] Abalos M, Bayona JM. Application of gas chromatography coupled to chemical ionisation mass spectrometry following headspace solid-phase microextraction for the determination of free volatile fatty acids in aqueous samples. J Chromatogr A. 2000;891(2):287–94. 11043789 10.1016/s0021-9673(00)00655-5

[38] McCullagh J, Probert F. New analytical methods focusing on polar metabolite analysis in mass spectrometry and NMR-based metabolomics. Curr Opin Chem Biol. 2024;80: 102466. 38772215 10.1016/j.cbpa.2024.102466

[39] Peng Y, Zhang Z, He L, Li C, Liu M. NMR spectroscopy for metabolomics in the living system: recent progress and future challenges. Anal Bioanal Chem. 2024;416(9):2319–34. 38240793 10.1007/s00216-024-05137-8 PMC10950998

[40] Szeremeta M, Pietrowska K, Niemcunowicz-Janica A, Kretowski A, Ciborowski M. Applications of metabolomics in forensic toxicology and forensic medicine. Int J Mol Sci. 2021;22(6):3010. 33809459 10.3390/ijms22063010 PMC8002074

[41] Zhou J, Yin Y. Strategies for large-scale targeted metabolomics quantification by liquid chromatography–mass spectrometry. Analyst. 2016;141(23):6362–73. 27722450 10.1039/c6an01753c

[42] Dudley E, Yousef M, Wang Y, Griffiths WJ. Targeted metabolomics and mass spectrometry. Adv Protein Chem Struct Biol. 2010;80:45–83. 21109217 10.1016/B978-0-12-381264-3.00002-3

[43] Schrimpe-Rutledge AC, Codreanu SG, Sherrod SD, McLean JA. Untargeted metabolomics strategies-challenges and emerging directions. J Am Soc Mass Spectrom. 2016;27(12):1897–905. 27624161 10.1007/s13361-016-1469-y PMC5110944

[44] Jeppesen MJ, Powers R. Multiplatform untargeted metabolomics. Magn Reson Chem. 2023;61(12):628–53. 37005774 10.1002/mrc.5350 PMC10948111

[45] Broeckling CD, Beger RD, Cheng LL, Cumeras R, Cuthbertson DJ, Dasari S, Davis WC, Dunn WB, Evans AM, Fernandez-Ochoa A, Gika H, Goodacre R, Goodman KD, Gouveia GJ, Hsu PC, Kirwan JA, Kodra D, Kuligowski J, Lan RS, Monge ME, Moussa LW, Nair SG, Reisdorph N, Sherrod SD, Ulmer Holland C, Vuckovic D, Yu LR, Zhang B, Theodoridis G, Mosley JD. Current practices in LC–MS untargeted metabolomics: a scoping review on the use of pooled quality control samples. Anal Chem. 2023;95(51):18645–54. 38055671 10.1021/acs.analchem.3c02924 PMC10753522

[46] Vo DK, Trinh KTL. Emerging biomarkers in metabolomics: advancements in precision health and disease diagnosis. Int J Mol Sci. 2024;25(23):13190. 39684900 10.3390/ijms252313190 PMC11642057

[47] Johnson CH, Ivanisevic J, Benton HP, Siuzdak G. Bioinformatics: the next frontier of metabolomics. Anal Chem. 2015;87(1):147–56. 25389922 10.1021/ac5040693 PMC4287838

[48] Ahmad P, Moussa DG, Siqueira WL. Metabolomics for dental caries diagnosis: past, present, and future. Mass Spectrom Rev. 2024;44:454. 38940512 10.1002/mas.21896

[49] DeBerardinis RJ, Keshari KR. Metabolic analysis as a driver for discovery, diagnosis, and therapy. Cell. 2022;185(15):2678–89. 35839759 10.1016/j.cell.2022.06.029 PMC9469798

[50] Proctor GB. The physiology of salivary secretion. Periodontolology 2000. 2016;70(1):11–25. 10.1111/prd.12116 26662479

[51] Yamamoto Y, Morozumi T, Takahashi T, Saruta J, To M, Sakaguchi W, Shimizu T, Kubota N, Tsukinoki K. Faster short-chain fatty acid absorption from the cecum following polydextrose ingestion increases the salivary immunoglobulin a flow rate in rats. Nutrients. 2020;12(6):1745. 32545166 10.3390/nu12061745 PMC7353249

[52] Pérez-Ros P, Navarro-Flores E, Julián-Rochina I, Martínez-Arnau FM, Cauli O. Changes in salivary amylase and glucose in diabetes: a scoping review. Diagnostics (Basel). 2021;11(3):453. 33800850 10.3390/diagnostics11030453 PMC8001770

[53] Qian J, Lu J, Huang Y, Wang M, Chen B, Bao J, Wang L, Cui D, Luo B, Yan F. Periodontitis salivary microbiota worsens colitis. J Dent Res. 2022;101(5):559–68. 34796773 10.1177/00220345211049781

[54] Sugimoto M. Salivary metabolomics for cancer detection. Expert Rev Proteomics. 2020;17(9):639–48. 33140667 10.1080/14789450.2020.1846524

[55] Ye D, Liu Y, Li J, Zhou J, Cao J, Wu Y, Wang X, Fang Y, Ye X, Zou J, Ma Q. Competitive dynamics and balance between Streptococcus mutans and commensal streptococci in oral microecology. Crit Rev Microbiol. 2024;51:532. 39132685 10.1080/1040841X.2024.2389386

[56] Zhang Y, Sun J, Lin CC, Abemayor E, Wang MB, Wong DT. The emerging landscape of salivary diagnostics. Periodontol 2000. 2016;70(1):38–52. 26662481 10.1111/prd.12099

[57] Zhang A, Sun H, Wang X. Saliva metabolomics opens door to biomarker discovery, disease diagnosis, and treatment. Appl Biochem Biotechnol. 2012;168(6):1718–27. 22971835 10.1007/s12010-012-9891-5

[58] Yang EC, Tan MT, Schwarz RA, Richards-Kortum RR, Gillenwater AM, Vigneswaran N. Noninvasive diagnostic adjuncts for the evaluation of potentially premalignant oral epithelial lesions: current limitations and future directions. Oral Surg Oral Med Oral Pathol Oral Radiol. 2018;125(6):670–81. 29631985 10.1016/j.oooo.2018.02.020 PMC6083875

[59] Vidas I, Delajlija M, Temmer-Vuksan B, Stipetić-Mravak M, Cindrić N, Maricíć D. Examining the secretor status in the saliva of patients with oral pre-cancerous lesions. J Oral Rehabil. 1999;26(2):177–82. 10080317 10.1046/j.1365-2842.1999.00314.x

[60] Kashyap B, Hyvärinen E, Laitinen I, Kullaa AM. Salivary metabolomics in patients with oral lichen planus: a preliminary study based on NMR spectroscopy. Clin Oral Investig. 2024;28(1):103. 38236502 10.1007/s00784-023-05389-1 PMC10796579

[61] Wang X, Liu L, Du Q, Sun Z, Yue E, Xue P, Zhao H. Human saliva metabolome for oral lichen planus biomarker identification. Recent Pat Anticancer Drug Discov. 2021;16(3):417–25. 33655848 10.2174/1574892816666210224160120

[62] Yan L, Xu J, Lou F, Dong Y, Lv S, Kang N, Luo Z, Liu Y, Pu J, Zhong X, Ji P, Xie P, Jin X. Alterations of oral microbiome and metabolic signatures and their interaction in oral lichen planus. J Oral Microbiol. 2024;16(1):2422164. 39498115 10.1080/20002297.2024.2422164 PMC11533246

[63] Yan SK, Wei BJ, Lin ZY, Yang Y, Zhou ZT, Zhang WD. A metabonomic approach to the diagnosis of oral squamous cell carcinoma, oral lichen planus and oral leukoplakia. Oral Oncol. 2008;44(5):477–83. 17936673 10.1016/j.oraloncology.2007.06.007

[64] Zuo L, Chen Z, Chen L, Kang J, Shi Y, Liu L, Zhang S, Jia Q, Huang Y, Sun Z. Integrative analysis of metabolomics and transcriptomics data identifies prognostic biomarkers associated with oral squamous cell carcinoma. Front Oncol. 2021;11: 750794. 34692531 10.3389/fonc.2021.750794 PMC8529182

[65] Yang E, Wang X, Gong Z, Yu M, Wu H, Zhang D. Exosome-mediated metabolic reprogramming: the emerging role in tumor microenvironment remodeling and its influence on cancer progression. Signal Transduct Target Ther. 2020;5(1):242. 33077737 10.1038/s41392-020-00359-5 PMC7572387

[66] Chen X, Yu D. Metabolomics study of oral cancers. Metabolomics. 2019;15(2):22. 30830419 10.1007/s11306-019-1483-8

[67] Hu S, Wang J, Ji EH, Christison T, Lopez L, Huang Y. Targeted metabolomic analysis of head and neck cancer cells using high performance ion chromatography coupled with a Q exactive HF mass spectrometer. Anal Chem. 2015;87(12):6371–9. 25973679 10.1021/acs.analchem.5b01350

[68] Yan X, Yang M, Liu J, Gao R, Hu J, Li J, Zhang L, Shi Y, Guo H, Cheng J, Razi M, Pang S, Yu X, Hu S. Discovery and validation of potential bacterial biomarkers for lung cancer. Am J Cancer Res. 2015;5(10):3111–22. 26693063 PMC4656734

[69] Zhou J, Hu Z, Wang L, Hu Q, Chen Z, Lin T, Zhou R, Cai Y, Wu Z, Zhang Z, Yang Y, Zhang C, Li G, Zeng L, Su K, Li H, Su Q, Zeng G, Cheng B, Wu T. Tumor-colonized Streptococcus mutans metabolically reprograms tumor microenvironment and promotes oral squamous cell carcinoma. Microbiome. 2024;12(1):193. 39369210 10.1186/s40168-024-01907-9 PMC11452938

[70] Sun YW, Chen KM, Aliaga C, El-Bayoumy K. Metabolic reprogramming in saliva of mice treated with the environmental and tobacco carcinogen dibenzo[def, p]chrysene. Sci Rep. 2024;14(1):29517. 39604478 10.1038/s41598-024-80921-1 PMC11603290

[71] Alapati S, Fortuna G, Ramage G, Delaney C. Evaluation of metabolomics as diagnostic targets in oral squamous cell carcinoma: a systematic review. Metabolites. 2023;13(8):890. 37623834 10.3390/metabo13080890 PMC10456490

[72] Bel’skaya LV, Sarf EA, Loginova AI. Diagnostic value of salivary amino acid levels in cancer. Metabolites. 2023;13(8):950. 37623893 10.3390/metabo13080950 PMC10456731

[73] Ohshima M, Sugahara K, Kasahara K, Katakura A. Metabolomic analysis of the saliva of Japanese patients with oral squamous cell carcinoma. Oncol Rep. 2017;37(5):2727–34. 28393236 10.3892/or.2017.5561

[74] Vimal J, George NA, Kumar RR, Kattoor J, Kannan S. Identification of salivary metabolic biomarker signatures for oral tongue squamous cell carcinoma. Arch Oral Biol. 2023;155: 105780. 37586141 10.1016/j.archoralbio.2023.105780

[75] de Sá AM, de Sá RN, Bandeira CM, Chagas JFS, Pascoal MBN, Nepomuceno G, da Silva MH, Alves MGO, Mendes MA, Dias M, Alves LAC, Almeida JD. Identification of possible salivary metabolic biomarkers and altered metabolic pathways in south American patients diagnosed with oral squamous cell carcinoma. Metabolites. 2021;11(10):650. 34677365 10.3390/metabo11100650 PMC8537096

[76] Lohavanichbutr P, Zhang Y, Wang P, Gu H, Nagana Gowda GA, Djukovic D, Buas MF, Raftery D, Chen C. Salivary metabolite profiling distinguishes patients with oral cavity squamous cell carcinoma from normal controls. PLoS ONE. 2018;13(9): e0204249. 30235319 10.1371/journal.pone.0204249 PMC6147497

[77] Wang Q, Gao P, Wang X, Duan Y. The early diagnosis and monitoring of squamous cell carcinoma via saliva metabolomics. Sci Rep. 2014;4:6802. 25354816 10.1038/srep06802 PMC4213796

[78] Sridharan G, Ramani P, Patankar S, Vijayaraghavan R. Evaluation of salivary metabolomics in oral leukoplakia and oral squamous cell carcinoma. J Oral Pathol Med. 2019;48(4):299–306. 30714209 10.1111/jop.12835

[79] Song X, Yang X, Narayanan R, Shankar V, Ethiraj S, Wang X, Duan N, Ni YH, Hu Q, Zare RN. Oral squamous cell carcinoma diagnosed from saliva metabolic profiling. Proc Natl Acad Sci USA. 2020;117(28):16167–73. 32601197 10.1073/pnas.2001395117 PMC7368296

[80] Ishikawa S, Hiraka T, Kirii K, Sugimoto M, Shimamoto H, Sugano A, Kitabatake K, Toyoguchi Y, Kanoto M, Nemoto K, Soga T, Tomita M, Iino M. Relationship between standard uptake values of positron emission tomography/computed tomography and salivary metabolites in oral cancer: a pilot study. J Clin Med. 2020;9(12):3958. 33297326 10.3390/jcm9123958 PMC7762245

[81] DeFelice BC, Fiehn O, Belafsky P, Ditterich C, Moore M, Abouyared M, Beliveau AM, Farwell DG, Bewley AF, Clayton SM, Archard JA, Pavlic J, Rao S, Kuhn M, Deng P, Halmai J, Fink KD, Birkeland AC, Anderson JD. Polyamine metabolites as biomarkers in head and neck cancer biofluids. Diagnostics (Basel). 2022;12(4):797. 35453845 10.3390/diagnostics12040797 PMC9024570

[82] Mikkonen JJW, Singh SP, Akhi R, Salo T, Lappalainen R, González-Arriagada WA, Ajudarte Lopes M, Kullaa AM, Myllymaa S. Potential role of nuclear magnetic resonance spectroscopy to identify salivary metabolite alterations in patients with head and neck cancer. Oncol Lett. 2018;16(5):6795–800. 30344764 10.3892/ol.2018.9419 PMC6176359

[83] Ishikawa S, Sugimoto M, Konta T, Kitabatake K, Ueda S, Edamatsu K, Okuyama N, Yusa K, Iino M. Salivary metabolomics for prognosis of oral squamous cell carcinoma. Front Oncol. 2021;11: 789248. 35070995 10.3389/fonc.2021.789248 PMC8769065

[84] Ishikawa S, Sugimoto M, Edamatsu K, Sugano A, Kitabatake K, Iino M. Discrimination of oral squamous cell carcinoma from oral lichen planus by salivary metabolomics. Oral Dis. 2020;26(1):35–42. 31602722 10.1111/odi.13209

[85] Tantray S, Sharma S, Prabhat K, Nasrullah N, Gupta M. Salivary metabolite signatures of oral cancer and leukoplakia through gas chromatography-mass spectrometry. J Oral Maxillofac Pathol. 2022;26(1):31–7. 35571322 10.4103/jomfp.jomfp_335_21 PMC9106257

[86] Antonelli R, Ferrari E, Gallo M, Ciociola T, Calciolari E, Spisni A, Meleti M, Pertinhez TA. The association between salivary metabolites and gingival bleeding score in healthy subjects: a pilot study. Int J Mol Sci. 2024;25(10):5448. 38791486 10.3390/ijms25105448 PMC11122368

[87] Kuboniwa M, Sakanaka A, Hashino E, Bamba T, Fukusaki E, Amano A. Prediction of periodontal inflammation via metabolic profiling of saliva. J Dent Res. 2016;95(12):1381–6. 27470067 10.1177/0022034516661142

[88] Wang LJ, Liu L, Ju W, Yao WX, Yang XH, Qian WH. 20 abnormal metabolites of Stage IV Grade C periodontitis was discovered by CPSI-MS. Pathol Oncol Res. 2022;28:1610739. 36567980 10.3389/pore.2022.1610739 PMC9768691

[89] Kim S, Kim HJ, Song Y, Lee HA, Kim S, Chung J. Metabolic phenotyping of saliva to identify possible biomarkers of periodontitis using proton nuclear magnetic resonance. J Clin Periodontol. 2021;48(9):1240–9. 34189748 10.1111/jcpe.13516

[90] Romano F, Meoni G, Manavella V, Baima G, Tenori L, Cacciatore S, Aimetti M. Analysis of salivary phenotypes of generalized aggressive and chronic periodontitis through nuclear magnetic resonance-based metabolomics. J Periodontol. 2018;89(12):1452–60. 29877582 10.1002/JPER.18-0097

[91] Liebsch C, Pitchika V, Pink C, Samietz S, Kastenmüller G, Artati A, Suhre K, Adamski J, Nauck M, Völzke H, Friedrich N, Kocher T, Holtfreter B, Pietzner M. The saliva metabolome in association to oral health status. J Dent Res. 2019;98(6):642–51. 31026179 10.1177/0022034519842853

[92] Andörfer L, Holtfreter B, Weiss S, Matthes R, Pitchika V, Schmidt CO, Samietz S, Kastenmüller G, Nauck M, Völker U, Völzke H, Csonka LN, Suhre K, Pietzner M, Kocher T. Salivary metabolites associated with a 5-year tooth loss identified in a population-based setting. BMC Med. 2021;19(1):161. 34256740 10.1186/s12916-021-02035-z PMC8278731

[93] Sakanaka A, Kuboniwa M, Katakami N, Furuno M, Nishizawa H, Omori K, Taya N, Ishikawa A, Mayumi S, Tanaka Isomura E, Shimomura I, Fukusaki E, Amano A. Saliva and plasma reflect metabolism altered by diabetes and periodontitis. Front Mol Biosci. 2021;8: 742002. 34589520 10.3389/fmolb.2021.742002 PMC8473679

[94] Ding J, Li J, Zhang C, Tan L, Zhao C, Gao L. High-throughput combined analysis of saliva microbiota and metabolomic profile in Chinese periodontitis patients: a pilot study. Inflammation. 2024;47(3):874–90. 38148454 10.1007/s10753-023-01948-6

[95] Wei Y, Shi M, Nie Y, Wang C, Sun F, Jiang W, Hu W, Wu X. Integrated analysis of the salivary microbiome and metabolome in chronic and aggressive periodontitis: a pilot study. Front Microbiol. 2022;13: 959416. 36225347 10.3389/fmicb.2022.959416 PMC9549375

[96] Buzalaf MAR, Ortiz AC, Carvalho TS, Fideles SOM, Araújo TT, Moraes SM, Buzalaf NR, Reis FN. Saliva as a diagnostic tool for dental caries, periodontal disease and cancer: is there a need for more biomarkers? Expert Rev Mol Diagn. 2020;20(5):543–55. 32223655 10.1080/14737159.2020.1743686

[97] Jacobson GR, Lodge J, Poy F. Carbohydrate uptake in the oral pathogen Streptococcus mutans: mechanisms and regulation by protein phosphorylation. Biochimie. 1989;71(9–10):997–1004. 2557096 10.1016/0300-9084(89)90103-x

[98] Abou Neel EA, Aljabo A, Strange A, Ibrahim S, Coathup M, Young AM, Bozec L, Mudera V. Demineralization–remineralization dynamics in teeth and bone. Int J Nanomed. 2016;11:4743–63. 10.2147/IJN.S107624 PMC5034904 27695330

[99] Musalem-Dominguez O, Montiel-Company JM, Ausina-Márquez V, Morales-Tatay JM, Almerich-Silla JM. Salivary metabolomic profile associated with cariogenic risk in children. J Dent. 2023;136: 104645. 37524196 10.1016/j.jdent.2023.104645

[100] Li Y, Yang Z, Cai T, Jiang D, Luo J, Zhou Z. Untargeted metabolomics of saliva in caries-active and caries-free children in the mixed dentition. Front Cell Infect Microbiol. 2023;13:1104295. 37082714 10.3389/fcimb.2023.1104295 PMC10110944

[101] Li K, Wang J, Du N, Sun Y, Sun Q, Yin W, Li H, Meng L, Liu X. Salivary microbiome and metabolome analysis of severe early childhood caries. BMC Oral Health. 2023;23(1):30. 36658579 10.1186/s12903-023-02722-8 PMC9850820

[102] Alvarenga-Brant R, Costa FO, Mattos-Pereira G, Esteves-Lima RP, Belém FV, Lai H, Ge L, Gomez RS, Martins CC. Treatments for burning mouth syndrome: a network meta-analysis. J Dent Res. 2023;102(2):135–45. 36214096 10.1177/00220345221130025

[103] Luo S, Lou F, Yan L, Dong Y, Zhang Y, Liu Y, Ji P, Jin X. Comprehensive analysis of the oral microbiota and metabolome change in patients of burning mouth syndrome with psychiatric symptoms. J Oral Microbiol. 2024;16(1):2362313. 38835338 10.1080/20002297.2024.2362313 PMC11149574

[104] Ye L, Dai Q, Hou F, Wu C, Qiu X, Yuan P, Chen F, Meng Y, Feng X, Jiang L. Salivary metabolomics of burning mouth syndrome: a cross-sectional study. Arch Oral Biol. 2022;144: 105552. 36279827 10.1016/j.archoralbio.2022.105552

[105] Moreau C, El Habnouni C, Lecron JC, Morel F, Delwail A, Le Gall-Ianotto C, Le Garrec R, Misery L, Piver E, Vaillant L, Lefevre A, Emond P, Blasco H, Samimi M. Salivary metabolome indicates a shift in tyrosine metabolism in patients with burning mouth syndrome: a prospective case-control study. Pain. 2023;164(3):e144–56. 35916738 10.1097/j.pain.0000000000002733

[106] Al-Hashimi I. The management of Sjögren’s syndrome in dental practice. J Am Dent Assoc. 2001;132(10):1409–17 (**quiz 60-1**). 11680356 10.14219/jada.archive.2001.0056

[107] Hou F, Cui Y, Ye L, Chen F, Wu C, Meng Y, Yuan P, Qiu X, Feng X, Jiang L. Metabolomic insights into idiopathic xerostomia: the central role of caffeine metabolism in salivary biochemistry. Arch Oral Biol. 2025;169: 106102. 39395317 10.1016/j.archoralbio.2024.106102

[108] Hynne H, Sandås EM, Elgstøen KBP, Rootwelt H, Utheim TP, Galtung HK, Jensen JL. Saliva metabolomics in dry mouth patients with head and neck cancer or Sjögren’s syndrome. Cells. 2022;11(3):323. 35159133 10.3390/cells11030323 PMC8833893

[109] LalueSanches M, Sforça ML, GuimarãesLoTurco E, Faber J, Smith RL, CarvalhodeMoraes LO. (1)H-NMR-based salivary metabolomics from females with temporomandibular disorders—a pilot study. Clin Chim Acta. 2020;510:625–32. 32791140 10.1016/j.cca.2020.08.006

[110] Adachi T, Kawanishi N, Ichigaya N, Sugimoto M, Hoshi N, Kimoto K. A preliminary pilot study: metabolomic analysis of saliva in oral candidiasis. Metabolites. 2022;12(12):1294. 36557332 10.3390/metabo12121294 PMC9786753

[111] Jo JK, Seo SH, Park SE, Kim HW, Kim EJ, Na CS, Cho KM, Kwon SJ, Moon YH, Son HS. Identification of salivary microorganisms and metabolites associated with halitosis. Metabolites. 2021;11(6):362. 34200451 10.3390/metabo11060362 PMC8226648

[112] Li Y, Wang D, Zeng C, Liu Y, Huang G, Mei Z. Salivary metabolomics profile of patients with recurrent aphthous ulcer as revealed by liquid chromatography–tandem mass spectrometry. J Int Med Res. 2018;46(3):1052–62. 29332424 10.1177/0300060517745388 PMC5972264

[113] Yatsuoka W, Ueno T, Miyano K, Enomoto A, Ota S, Sugimoto M, Uezono Y. Time-course of salivary metabolomic profiles during radiation therapy for head and neck cancer. J Clin Med. 2021;10(12):2631. 34203786 10.3390/jcm10122631 PMC8232617

[114] Zhou J, Hu H, Huang R. A pilot study of the metabolomic profiles of saliva from female orthodontic patients with external apical root resorption. Clin Chim Acta. 2018;478:188–93. 29291387 10.1016/j.cca.2017.12.046

[115] Song Z, Fang S, Guo T, Wen Y, Liu Q, Jin Z. Microbiome and metabolome associated with white spot lesions in patients treated with clear aligners. Front Cell Infect Microbiol. 2023;13:1119616. 37082715 10.3389/fcimb.2023.1119616 PMC10111054

[116] Ai JY, Smith B, Wong DT. Bioinformatics advances in saliva diagnostics. Int J Oral Sci. 2012;4(2):85–7. 22699264 10.1038/ijos.2012.26 PMC3412667

[117] Grootveld M, Page G, Bhogadia M, Hunwin K, Edgar M. Updates and original case studies focused on the NMR-linked metabolomics analysis of human oral fluids part III: implementations for the diagnosis of non-cancerous disorders, both oral and systemic. Metabolites. 2023;13(1):66. 36676991 10.3390/metabo13010066 PMC9864626

[118] Alamri MM, Williams B, Le Guennec A, Mainas G, Santamaria P, Moyes DL, Nibali L. Metabolomics analysis in saliva from periodontally healthy, gingivitis and periodontitis patients. J Periodontal Res. 2023;58(6):1272–80. 37787434 10.1111/jre.13183

[119] Ishikawa S, Sugimoto M, Kitabatake K, Tu M, Sugano A, Yamamori I, Iba A, Yusa K, Kaneko M, Ota S, Hiwatari K, Enomoto A, Masaru T, Iino M. Effect of timing of collection of salivary metabolomic biomarkers on oral cancer detection. Amino Acids. 2017;49(4):761–70. 28101653 10.1007/s00726-017-2378-5

[120] Deng Q, Wong HM, Peng S. Alterations in salivary profile in individuals with dental caries and/or obesity: a systematic review and meta-analysis. J Dent. 2024;151: 105451. 39505293 10.1016/j.jdent.2024.105451

[121] Aceña J, Stampachiacchiere S, Pérez S, Barceló D. Advances in liquid chromatography-high-resolution mass spectrometry for quantitative and qualitative environmental analysis. Anal Bioanal Chem. 2015;407(21):6289–99. 26138893 10.1007/s00216-015-8852-6

[122] Alonso-Moreno P, Rodriguez I, Izquierdo-Garcia JL. Benchtop NMR-based metabolomics: first steps for biomedical application. Metabolites. 2023;13(5):614. 37233655 10.3390/metabo13050614 PMC10223723

[123] Wang J, Christison TT, Misuno K, Lopez L, Huhmer AF, Huang Y, Hu S. Metabolomic profiling of anionic metabolites in head and neck cancer cells by capillary ion chromatography with Orbitrap mass spectrometry. Anal Chem. 2014;86(10):5116–24. 24766394 10.1021/ac500951v

[124] Suragimath G, Patil S, Suragimath DG. Salivaomics: a revolutionary non-invasive approach for oral cancer detection. Cureus. 2024;16(11): e74381. 39723315 10.7759/cureus.74381 PMC11669377

[125] Bahbah EI, Noehammer C, Pulverer W, Jung M, Weinhaeusel A. Salivary biomarkers in cardiovascular disease: an insight into the current evidence. FEBS J. 2021;288(22):6392–405. 33370493 10.1111/febs.15689

[126] Herrala M, Turunen S, Hanhineva K, Lehtonen M, Mikkonen JJW, Seitsalo H, Lappalainen R, Tjäderhane L, Niemelä RK, Salo T, Myllymaa S, Kullaa AM, Kärkkäinen O. Low-dose doxycycline treatment normalizes levels of some salivary metabolites associated with oral microbiota in patients with primary Sjögren’s syndrome. Metabolites. 2021;11(9):595. 34564411 10.3390/metabo11090595 PMC8470364

